# Chloroimidazolium Deoxyfluorination Reagent with H_2_F_3_^–^ Anion as a Sole Fluoride Source

**DOI:** 10.1021/acs.joc.4c00787

**Published:** 2024-07-15

**Authors:** Griša Prinčič, Blaž Omahen, Jan Jelen, Evelin Gruden, Gašper Tavčar, Jernej Iskra

**Affiliations:** † Department of Chemistry and Biochemistry, Faculty of Chemistry and Chemical Technology, University of Ljubljana, Večna pot 113, 1000 Ljubljana, Slovenia; ‡ Department of Inorganic Chemistry and Technology, “Jožef Stefan” Institute, Jamova cesta 39, 1000Ljubljana, Slovenia

## Abstract

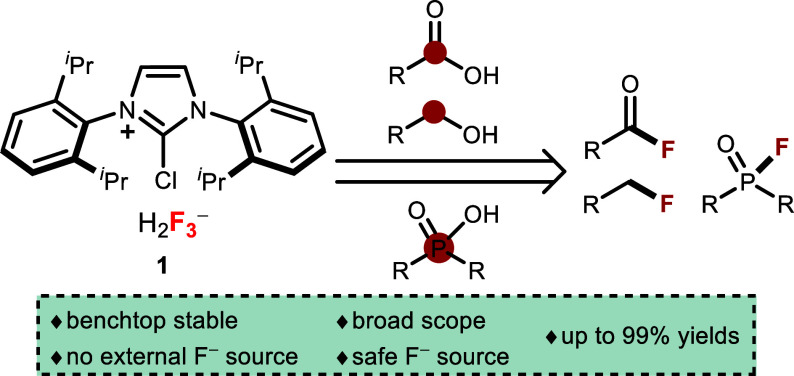

In the study, we introduce an air-stable NHC-based deoxyfluorination reagent ImCl[H_2_F_3_], offering a promising avenue for deoxyfluorination across various substrates. Reagent efficiently fluorinates benzyl alcohols, carboxylic acids, and P(V) compounds without external fluoride sources. A mechanistic study reveals a two-step process involving benzyl chloride as an intermediate, shedding light on the two-step reaction pathway. The Hammet plot provides insights into reaction mechanisms with different substrates, enhancing our understanding of this versatile deoxyfluorination method.

## Introduction

The introduction of carbon–fluorine bonds into organic compounds can bring about substantial alterations in their chemical and physical attributes when compared to their nonfluorinated alternatives, leading to profound improvement and metabolic stability of pharmaceuticals, drugs, and pesticides.^1^ This transformation primarily arises from the numerous advantageous properties conferred upon these molecules by the fluorine atoms in the structure. These benefits encompass the ability to lower the p*K*_a_ of adjacent functional groups or induce conformational changes to the molecule, making it less accessible for enzymatic degradation.^1f,2^ Consequently, fluorine has become a critical component in approximately 20% of pharmaceuticals and more than 50% of agrochemicals sanctioned by the Food and Drug Administration in 2021 alone.^3^ To fully exploit the potential of this element, various fluorine-containing functional groups have been incorporated into drug targets.^4^

Beyond its capacity to modify chemical properties, fluorine can exert a significant influence on the reactivity of compounds. For example, acyl fluorides, distinct from acyl chlorides, serve as invaluable synthons due to their exceptional balance between stability and reactivity.^5^ Additionally, acyl fluorides can function as versatile synthetic equivalents for various moieties like “Ar–CO”,^6^ “Ar”,^7^ and “F″,^8^ but also act as an important precursor to esters^9^ and amides.^10^ Furthermore, there is a growing appreciation for phosphorus–fluorine bonds, particularly those grounded in P(V) compounds. This class of molecules has assumed pivotal roles as enzyme inhibitors, mechanistic probes, and organocatalysts in diverse synthetic processes.^11^

To gain access to organofluorine compounds, methods involve deoxyfluorination of alcohols, carboxylic acids, and aldehydes with reagents such as DAST, DeoxoFluor, PyFluor,^12^ and the most recently developed NHC-based reagents like AlkylFluor^13^ or PhenoFluor, as well as some recently developed methods for deoxyfluorination of organoboron and olefin compounds with visible light.^14^ The latter require mild reaction conditions and can tolerate a plethora of different functional groups while still providing high yield deoxyfluorination reactions. Despite their outstanding properties, most deoxyfluorination reagents require external sources of fluoride like CsF, KF, or halide abstractors like Ag_2_CO_3_ in large, up to 10-fold access.^13,15^ Not only that, PhenoFluorMix and AlkylFluor are specific for either alcohols or phenols; PhenoFluor, specific for both types of substrates, is sensitive to moisture and long-term storage.

There is still room to improve on the current state of the art. In light of these observations, we used a new, air-stable NHC-based deoxyfluorination reagent ImCl[H_2_F_3_] **1** that bears a H_2_F_3_^–^ polyfluoride anion in its structure (Table 1).^16^ ImCl[H_2_F_3_] **1** is completely air-stable and does not decompose upon storage. Also, most NHC reagents require inert conditions, while **1** is prepared in aqueous media under ambient conditions using hypochlorite as a chlorinating agent. The efficacy of **1** was demonstrated previously on deoxyfluorination of electron-deficient phenols.^17^ Herein, we report the study on the reactivity of **1** for deoxyfluorination of benzyl alcohols, carboxylic acids, and organophosphates like phosphinic acids and phosphates as well as organosulfuric acids without any addition of an external source of fluoride.

**Table 1:**
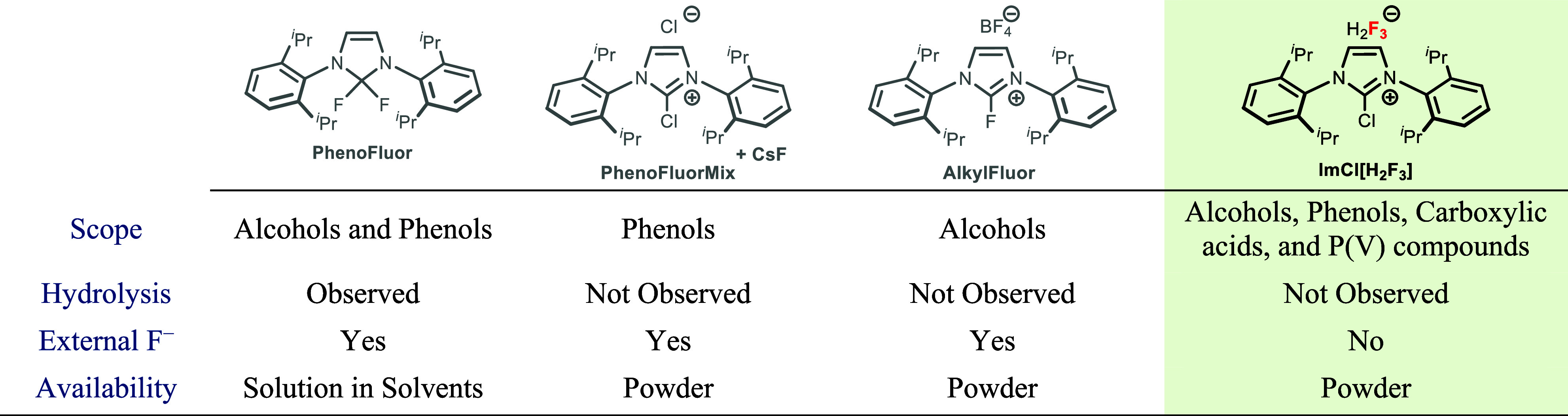
Comparison of NHC-Based Deoxyfluorination Reagents

## Results and Discussion

We first turned our attention toward deoxyfluorination of benzyl alcohols. Initially, we chose 4-*tert*-butyl benzyl alcohol **2b** as a model substrate for the deoxyfluorination reaction to the corresponding 4-*tert*-butyl benzyl fluoride **3b**. We found that a combination of 2 equiv of **1** and 4 equiv of 2-*tert*-butyl-1,1,3,3-tetramethyl guanidine (BTMG) in acetonitrile at 100 °C for 3 h provided **3b** in a 76% ^1^H NMR yield. Selected variations of reaction conditions are given in Table 2 (the extensive list of reaction conditions is provided in the Supporting Information in Table S-1). Reactions run without the presence of BTMG confirm that the base is indeed needed to obtain target product **3b**. Using different bases such as diisopropylethylamine (DIPEA), 1,8-diazabicyclo[5.4.0]undec-7-ene (DBU), or carbonate-based bases like Cs_2_CO_3_ significantly lowered the yield and afforded **3b** in 10–45% yield. Acetonitrile alternatives like hexamethylphosphoramidite (HMPA), tetrahydrofuran (THF), dioxane, or dimethoxyethane (DME) were less effective. Results indicate that the specific combination of the organic base and solvent gives the best yield, which is also the case for carboxylic acids. Running the reaction at lower temperatures also diminished the yield significantly. Reaction times were also investigated (see Table S-1 for more details). In the first hour of the reaction, the yields of **3b** from **2b** dramatically increase and reach a plateau after 3 h.

**Table 2:** Optimization of Reaction Conditions for Conversion of **2b** to **3b**^a^

entry	deviation from standard conditions	yield (%)^b^
1	none	76
2	no base	<10
3	DBU, DIPEA, Cs_2_CO_3_, Pyridine, Et_3_N base	10–45
4	DME, THF, dioxane, DMAC, HMPA, PhMe instead of MeCN	14–70
5	temperature rt −80	<1–68

a Standard reaction conditions: **2b** (0.1 mmol), ImCl[H_2_F_3_] (0.2 mmol, 2.0 equiv), and BTMG (0.4 mmol, 4.0 equiv) in MeCN (1 mL, 0.1M) at 100 °C for 3 h.

b Determined with ^1^H NMR spectroscopy with naphthalene as an internal standard.

**Scheme 1:**
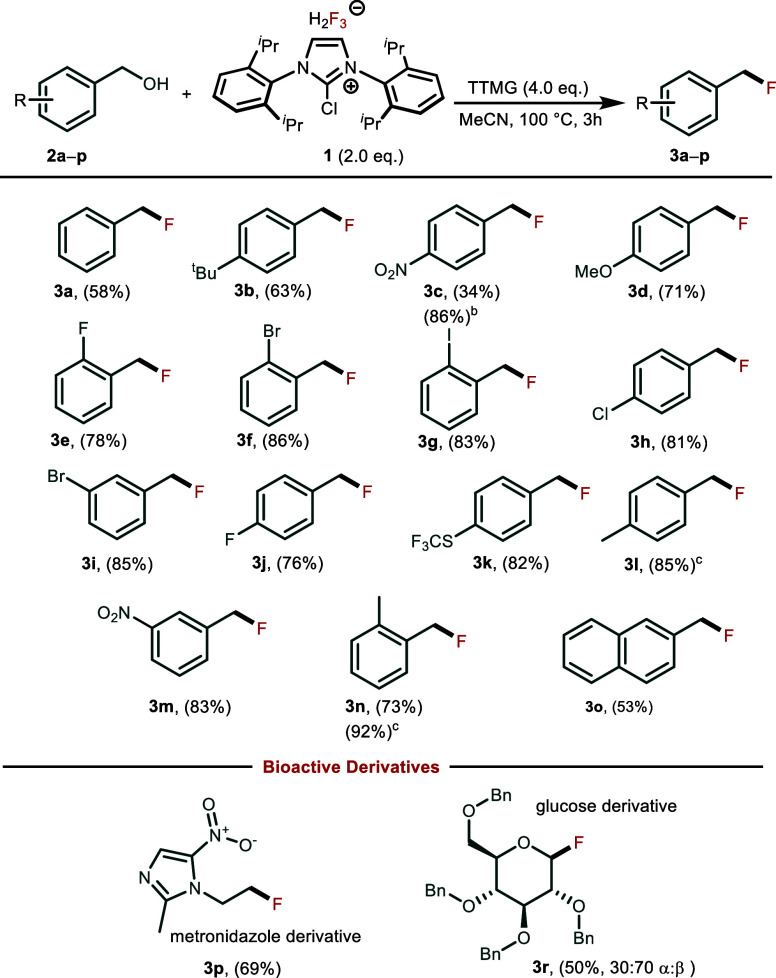
Synthesis of Benzyl Fluorides from Benzyl Alcohols **2** Reaction conditions: **2** (0.1 mmol), ImCl[H_2_F_3_] **1** (0.2 mmol, 2.0 equiv) and BTMG (0.4 mmol, 4.0 equiv), MeCN (1 mL, 1 M), 100 °C, 3 h. Yields were determined by quantitative ^1^H NMR with naphthalene as an internal standard. 0.2 mmol of AgOTf was added to the reaction mixture. Reaction time was prolonged to 18 h.

We then turned our attention to study the substrate scope under optimized reaction conditions (Scheme 1). All 4-substituted benzyl alcohols bearing electron-donation groups like -OMe and -Me yielded fluorinated products **3** in moderate to good yields (63–85%). Substrates with slightly electron-withdrawing groups like -F, -Cl, and -Br also reacted with good yields (81–86%). Substrates with strongly electron-withdrawing groups like 4-NO_2_**2c** initially gave a poor yield of 34%. The addition of 2 equiv of silver trifluoromethanesulfonate (AgOTf) to the reaction, however, drastically improved the yield to 86%. Prolongation of reaction time to 18 h also increased the yields of 4-Me **2l** and 2-Me **2n** substituted benzyl alcohols but have proven to be ineffective for other substrates. Naphthyl derivative **2o** afforded the corresponding fluoride in 53% yield, demonstrating the diminished reactivity due to bulkiness of the naphthyl substituent. To demonstrate the effectiveness of the method, we also deoxyfluorinated 1 and 2° alkyl substrates with a more complex and bioactive nature metronidazole **2p** and glucose **2r** in 69 and 50% yield, respectively.

Under exemplary reaction conditions, secondary and tertiary alkyl alcohols reacted poorly and gave fluoroalkanes in the mixture of their respective alkenes. We used 1-octanol as an example of a primary alkyl alcohol. The yield of 1-fluorooctane was 10% with the rest being identified as 1-octene. 2-Octanol gave similar results with most of the product being 1- and 2-octene. As an example of tertiary alcohols, we used 2-phenyl-2-propanol, where only 6% yield was determined for its fluorinated product, 87% was the starting material, and 7% was the 2-alkene product.

**Scheme 2:**
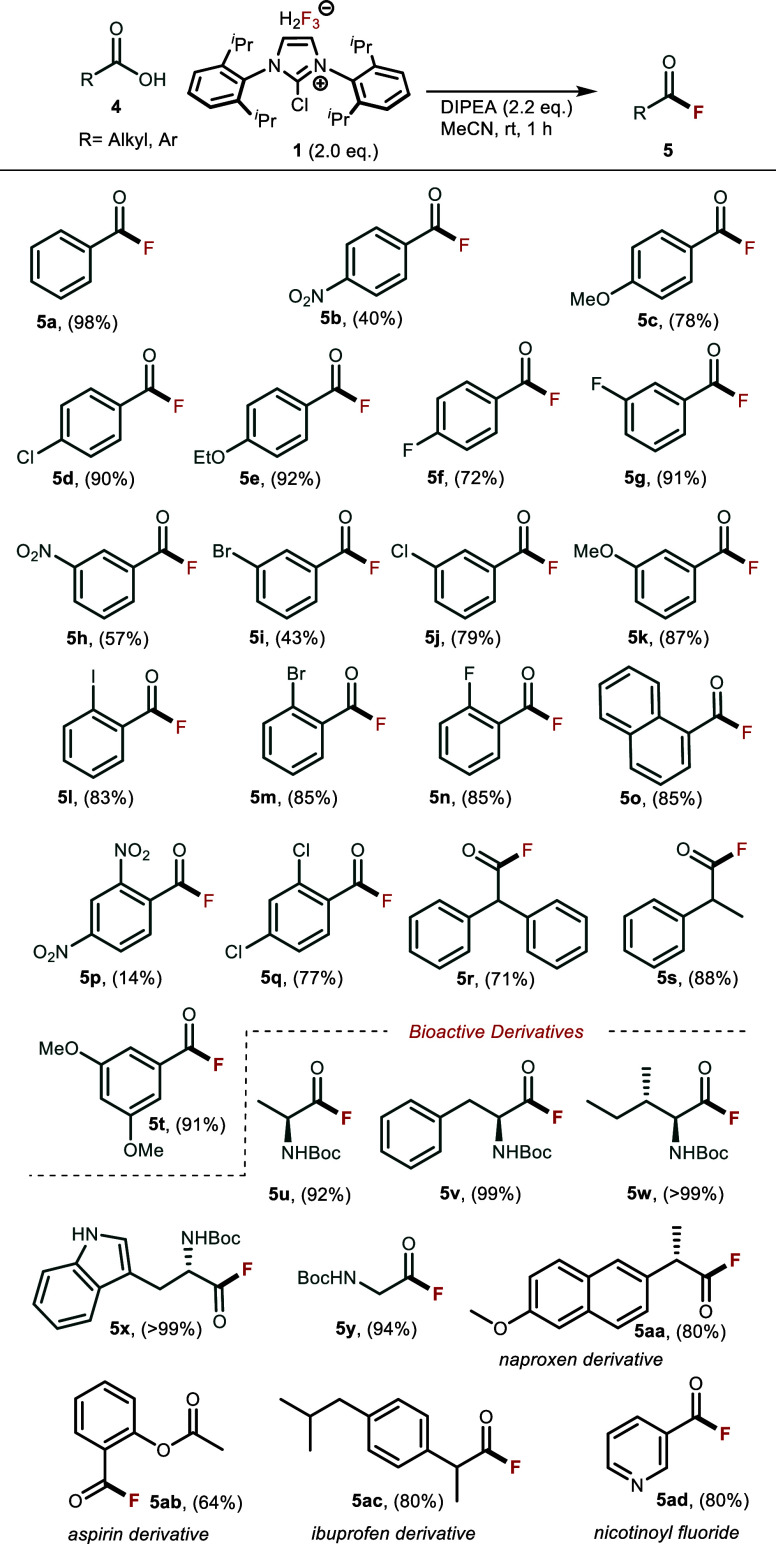
Synthesis of Acyl Fluorides from Carboxylic Acids with **1** Reaction conditions: carboxylic acid **4** (0.1 mmol), reagent **1** (0.11 mmol, 1.1 equiv), and DIPEA (0.22 mmol, 2.2 equiv) in MeCN (1 mL, 0.1 M) were stirred at room temperature for 1 h. Yields were determined by quantitative ^1^H NMR with naphthalene as an internal standard.

Since carboxylic acids react much faster and the resulting acyl fluorides are more reactive and unstable than benzyl fluorides, we decided to optimize reaction conditions for deoxyfluorination of carboxylic acids with **1** at room temperature (Scheme 2). We used benzoic acid **4a** as a model substrate and found that 1 equiv of **1**, 2.2 equiv access of DIPEA as a base in acetonitrile gave 98% conversion of **4a** to **5a** (for extended screening of reaction conditions, see Supporting Information Table S-2). Benzoic acids with electron-donating groups such as methoxy substituted derivatives were the most reactive with the highest yield observed with 3,5-dimethoxybenzoic acid **4t** (91%) and ethoxy derivative **4e**. 2-substituted derivatives **4l**, **4m,** and **4n**, despite their steric encumbrance, reacted well and gave good to excellent yields (77–85%). Naphthyl derivative **4o** as well as diphenyl **4r** and phenyl-methyl **4s** substituted benzoic acids also reacted well with 71–88% yields, demonstrating that steric effects have little to no influence on transformation. The opposite trend was seen in benzoic acids with electron-withdrawing groups. These were less reactive and gave their respective fluorides in 14–40% yields. The drop in yield is seen especially in double-substituted 2,4-dinitrobenzoic acid, where the electron-withdrawing nature of two NO_2_ groups significantly affected the conversion and gave 2,4-dinitrobenzyl fluoride **5p** in only 14% yield.

Alongside benzoic acids, we applied the method to Boc-protected amino acids **4u**–**y** and converted them to corresponding acyl fluorides **5u**–**y**, important synthons for amide and peptide preparation. Yields of their respective fluorides ranged from 92% to quantitative in the case of the more nonpolar isoleucine derivatives **4u**–**w**. Deoxyfluorination of some noncorticosteroid anti-inflammatory drugs **4aa–ad** (NSAIDs) like Naproxen, Aspirin, and Ibuprofen yielded their aliphatic acyl fluoride derivatives in good to excellent yields of 64–80%.

**Scheme 3:**
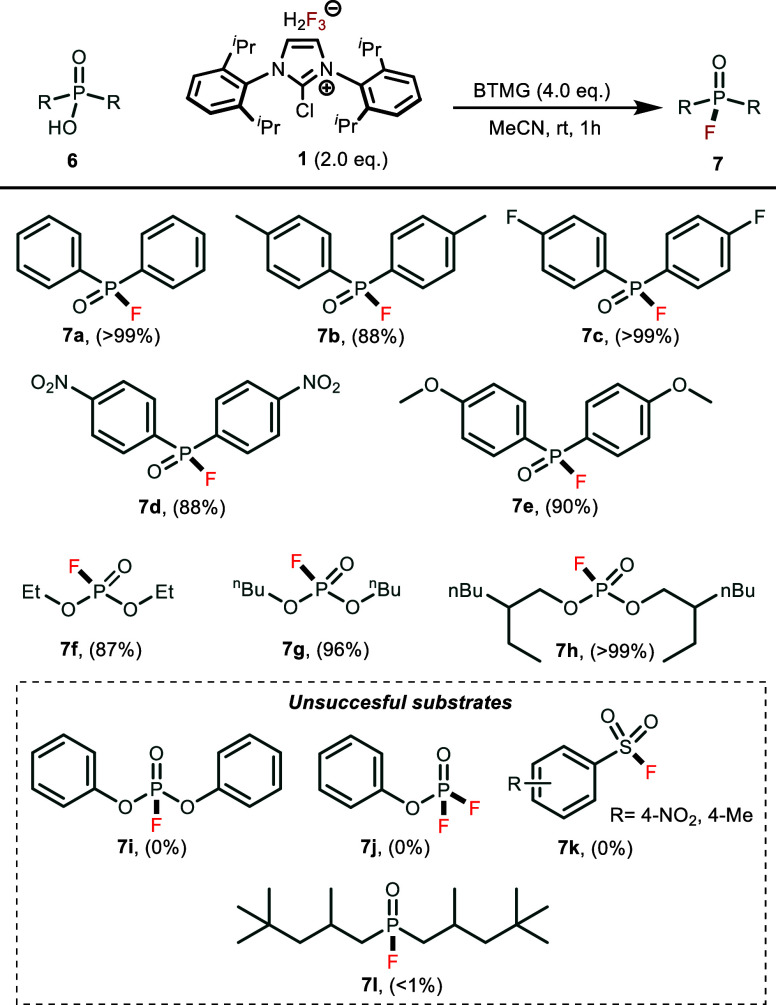
Synthesis of P(V) Compounds from Phosphonic Acids and Phosphates Reaction conditions: Phosphinic acid or phosphate **6** (0.1 mmol), reagent **1** (0.1 mmol, 1.0 equiv), and BTMG (0.11 mmol, 1.1 equiv) in MeCN (1 mL, 1 M) were stirred at room temperature for 1 h. Yields were determined by quantitative ^1^H NMR with naphthalene as an internal standard.

Last but not least, we used **1** to prepare phosphonic fluorides and fluorophosphates. Initially, we used the same reaction conditions as for carboxylic acids; however, conversions after 24 h at room temperature were incomplete. After optimization, we found that by increasing **1** to 2 equiv and use BTMG as a base, we were able to achieve quantitative conversions within 1 h at room temperature (for extended screening of reaction conditions see Table S-3). Diphenylphosphinic acids with both electron-withdrawing −NO_2_ group and electron-donating −OMe group reacted to produce their corresponding phosphonic fluorides in excellent to quantitative yields (88– <99%). Dialkyl hydrogen phosphates with short alkyl chains **6f** and **6g** gave excellent yields under these reaction conditions (87–96%). Interestingly, phenyl and diphenylphosphates **6j** and **6i**, dialkylphosphinates **7l** as well as benzenesulfonic acid **6k** did not react and no traces of the fluorinated products were observed during analysis (Scheme 3).

Ritter and co-workers suggest a formation of 2-aryloxyimdiazolium and a tetrahedral intermediate during the deoxyfluorination of phenols with PhenoFluor that then rearranges via a concerted transition state producing aryl fluorides and 2-imidazolone as a side product.^15b^ No such stable intermediates were observed with benzyl alcohols (NMR and HRMS analyses).

**Scheme 4:**
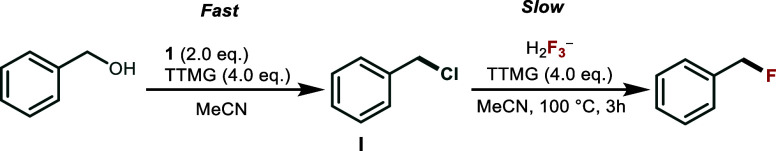
Proposed Reaction Pathway for Deoxyfluorination with **1**

A mechanistic study of the deoxyfluorination of benzyl and also alkyl alcohols shows that the reaction proceeds in two steps. In the first step, the chloride atom is transferred to the benzyl site, forming the corresponding benzyl chloride **I** and imidazolone. In the second step, benzyl fluoride is formed from benzyl chloride **I** and fluoride anion derived from H_2_F_3_^–^ (Scheme 4). Benzyl chloride **I** was the sole product when reactions were run at ambient temperatures (see Table S-4) and only elevated temperatures resulted in the formation of the corresponding fluoride. Intermediate product **I** was isolated in an independent experiment between benzyl alcohol **3a** and fluorinating reagent **1** at room temperature.

**Caution!** Organophosphates are potentially extremely toxic! Fluoroorganophosphates are known neurotoxins! Reactions were performed in a fume hood, and residues were diluted with a NaOH solution and disposed of properly!

The Hammet plot with differently substituted benzyl alcohols suggests that the chlorination step of the reaction proceeds via the S_N_2 reaction mechanism with an observed Hammet value of ρ = 0.51 (see Graph S-1). The chlorinated intermediary product was, however, not observed in the case of carboxylic acids, where deoxyfluorination seems to proceed in one step and yields acyl fluorides directly. The Hammet constant of ρ = −2.6 reveals that the reaction loses negative charge, suggesting a carboxylate attack on the 2-chloroimdiazolium ion as a rate-determining step (see Graph S-2). The formed 2-carboxyimidazolium intermediate then undergoes a rapid conversion to the corresponding acyl fluoride and imidazolone side product. The Hammet correlation plot for substituted diphenylphosphinic acids with ρ = −2.0 suggests the same reaction mechanism as with carboxylic acids (see Graph S-3).

## Conclusions

In conclusion, the newly developed NHC-based reagent with H_2_F_3_ anion in its structure has shown to be an efficient and stable reagent for deoxyfluorination of a wide range of different substrates like benzylic and aliphatic alcohols, carboxylic acids, phosphinic acids, and phosphates without the need for an external fluoride source. Alongside standard substrates, the method was also applied to amino acid derivatives and commonly prescribed drugs as bioactive compounds. Optimized reaction conditions give benzylic and aliphatic fluorides from alcohols with good to excellent yields. In the case of carboxylic acids, the reagent loading was lowered to 1 equiv, and yields of their respective acyl fluorides ranged to quantitative in 1 h at standard conditions. Phosphinic acids and phosphates also reacted well and afforded P(V) fluorides with quantitative yields. The mechanism of deoxyfluorination of benzyl alcohols proceeds via two sequential steps, where benzyl chloride is formed in the first step and is later transformed into the corresponding fluoride via another S_N_2 reaction with fluoride to form the H_2_F_3_^–^ anion.

## Data Availability

The data underlying this study are available in the published article and its Supporting Information.

## Supporting Information Available

Optimization studies, Hammett plots, and details of all experimental procedures and spectroscopic data (PDF)
