# Supplementary material for: Chloroimidazolium Deoxyfluorination Reagent with H2F3– Anion as a Sole Fluoride Source
Source: J Org Chem. 2024 Jul 15;89(15):10557–61. doi: 10.1021/acs.joc.4c00787 (PMC11301684; doi:10.1021/acs.joc.4c00787)
Supplement: Supplementary file 1 — jo4c00787_si_001.pdf [file jo4c00787_si_001.pdf]

# Chloroimidazolium Deoxyfluorination Reagent with $H_2F_3^-$ Anion as a Sole Fluoride Source

Griša Prinčič[a], Blaž Omahen[a], Jan Jelen[b], Evelin Gruden[b], Gašper Tavčar[b], Jernej Iskra[a]*

[a]Department of Chemistry and Biochemistry, University of Ljubljana, Faculty of Chemistry and Chemical Technology, Večna pot 113, 1000 Ljubljana, Slovenia.

[b]Department of Inorganic Chemistry and Technology, "Jožef Stefan" Institute, Jamova cesta 39, Ljubljana, Slovenia.

E-mail: *jernej.iskra@fkkt.uni-lj.si*

# Supporting Information

## Table of Contents

General Experimental Section ........ S2

Abbreviations ........ S2

Instrumentation and materials ........ S2

Experimental Procedures ........ S2

General procedures for preparation of fluorides with reagent 1 ........ S2

Mechanistic study experiments ........ S4

Hammet correlation experimental details ........ S5

Spectroscopic characterization ........ S8

Spectroscopic data for benzyl fluorides ........ S8

Spectroscopic data for benzoyl fluorides ........ S13

Spectroscopic data for amino acid fluorides and biologically active compounds ........ S19

Spectroscopic data for fluoro phosphates and phosphinates ........ S21

# General Experimental Section

## Abbreviations

**BTMG** = *tert*-butyl tetramethyl guanidine, **DBU** = 1,8-Diazabicyclo[5.4.0]undec-7-ene, **DME** = 1,2-dimethoxyethane, **ImCl[$H_2F_3$]** = 2-chloro-1,3-bis(2,6-diisopropylphenyl)-1*H*-imidazol-3-ium dihydrogen trifluoride salt.

## Instrumentation and materials

All chemicals, materials and solvents were purchased from commercial sources and used without further purification unless otherwise noted. Solvents (PhMe, $Et_2O$, MeCN) were distilled over sodium wire and then stored over 3 Å molecular sieves (20% v/v) for at least 72 hours prior to use. $^1H$, $^{19}F$, $^{31}P$ and $^{13}C\{^1H\}$ NMR spectra were recorded on a Bruker Avance III 500 MHz NMR spectrometer (500 MHz for $^1H$, 471 MHz for $^{19}F$ and 126 MHz for $^{13}C$) and Bruker Ascend 600 MHz NMR spectrometer. Chemical shifts were reported as delta scale in ppm relative to $CDCl_3$ (residual $CHCl_3$ $\delta$ = 7.26 ppm for $^1H$, 77.16 ppm for $^{13}C$). $CCl_3F$ for $^{19}F$ ($\delta$ = 0.00 ppm) and $H_3PO_4$ for $^{31}P$ ($\delta$ = 0.00 ppm). Proton magnetic resonance spectra ($^1H$ NMR) were recorded with 10 second delay between scans to ensure accurate integration. High-resolution Atmospheric Pressure Chemical Ionization Time-of-Flight Mass (HR-APCI-TOF-MS) spectra were recorded on an Agilent 6224 Accurate Mass TOF LC/MS System. Reagent **1** was prepared according to literature procedure and stored in ambient conditions. Prior to use **1** was dried at 70 °C under vacuum.[1] Phosphinates **6b–e** were prepared according to modified procedure.[2]

# Experimental Procedures

## General procedures for preparation of fluorides with reagent 1

### Benzyl fluorides; General procedure A

Benzyl alcohol **2a–p** (0.1 mmol) and naphthalene (1.0 eq., 0.1 mmol as internal standard) were dissolved in dry MeCN (1 mL, 0.1 M) in a glass vial. Reagent **1** (2.0 eq., 97 mg, 0.2 mmol) was added and the solution was stirred for 1 min until full dissolution. BTMG (4.0 eq., 0.4 mmol, 68.5 mg) was added in one portion, the vial was sealed with a Teflon lined stopper and heated in an aluminum heating block at 100 °C for 3 h. After that, the reaction was let to cool to room temperature and 10 µL was transferred to a dry NMR tube and 500 µL of $CDCl_3$ was added.

### Acyl fluorides; General procedure B

Carboxylic acids **4a–ad** (0.1 mmol) and naphthalene (1.0 eq., 0.1 mmol as internal standard) were dissolved in dry MeCN (1 mL, 0.1 M) in a glass vial. Reagent **1** (1.1 eq., 48.3 mg, 0.1 mmol) was added and the solution was stirred for 1 min until full dissolution. DIPEA (2.0 eq., 0.2 mmol, 26 mg) was added in one portion, the vial was sealed with a Teflon lined stopper and stirred at rt for 1 h. After that, 10 µL was transferred to a dry NMR tube and 500 µL of $CDCl_3$ was added.

### Fluorophosphates and phosphinates; General procedure C

Phosphates or phosphinates **6a–k** (0.1 mmol) and naphthalene (1.0 eq., 0.1 mmol as internal standard) were dissolved in dry MeCN (1 mL, 0.1 M) in a glass vial. Reagent **1** (2.0 eq., 97 mg, 0.2 mmol) was

added and the solution was stirred for 1 min until full dissolution. BTMG (4.0 eq., 0.4 mmol, 68.5 mg) was added in one portion, the vial was sealed with a Teflon lined stopper and stirred at rt for 1 h. After that, 10 µL was transferred to a dry NMR tube and 500 µL of $CDCl_3$ was added.

**Caution!** Organo phosphates are potentially extremely toxic! Fluoroorgano phosphates are known neurotoxins! Reactions were performed in a fume hood and residues were diluted with NaOH solution and disposed of properly!

**Larger scale (1 mmol) synthesis of 4-methylbenzyl fluoride 3l**

4-methylbenzyl alcohol **2l** was dissolved in dry MeCN (10 mL, 0.1 M) in a Teflon™ reactor. Reagent **1** (2.2 eq., 483 mg, 2.2 mmol) was added and the solution was stirred for 1 min until full dissolution. DIPEA (6.0 eq., 6 mmol, 774 mg) was added in one portion, the reactor was heated in an oil bath at 140 °C for 24 h. The product was isolated by the following procedure: the solvent was evaporated, and the residue was dissolved in *n*-pentane. The product was purified by silica gel column flash chromatography using *n*-pentane as eluent (mixture of 4-methylbenzyl fluoride **3l** and 4-methylbenzyl chloride 70:30, clear liquid). The ratio was determined with $^{1}$H NMR. The mass of the product after evaporation of the solvent was 100 mg (78% yield).

**Table S- 1:** Optimization of reaction conditions for fluorination of benzyl alcohols with reagent **1**

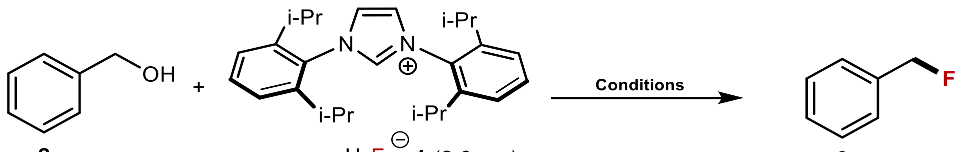


| Entry | Solvent | Base (eq.) | Conditions (T/t) | Reagent (eq.) | Yield (%) |
|---|---|---|---|---|---|
| 1 | PhMe | DIPEA | 140 °C/2 h | 1.5 | 5 |
| 2 | PhMe | DBU | 140 °C/2h | 2.0 | 45 |
| 3 | PhMe | DBU | 140 °C/on | 2.0 | 70 |
| 4 | MeCN | DIPEA | 80 °C/2h | 1.5 | < 1 |
| 5 | MeCN | DIPEA | 140 °C/ 2h | 1.5 | 12 |
| 6 | MeCN | DIPEA | 140 °C/on | 2.0 | 66 |
| 7 | DME | DIPEA | 140 °C/on | 2.0 | 23 |
| 8 | THF | DIPEA | 140 °C/on | 2.0 | 14 |
| 9 | Dioxane | DIPEA | 140 °C/on | 2.0 | 10 |
| 10 | DMAC | DIPEA | 140 °C/on | 2.0 | 28 |
| 11 | MeCN | TMG | 140 °C/on | 2.0 | 33 |
| 12 | MeCN | $Cs_2CO_3$ | 140 °C/on | 2.0 | 10 |
| 13 | MeCN | BTMG | 100 °C/3 h | 2.0 | 68 |
| 14 | HMPA | DIPEA | 140 °C/24 h | 2.0 | 2 |

on = overnight

**Table S- 2:** Optimization of reaction conditions for fluorination of benzoic acids with **reagent** 1

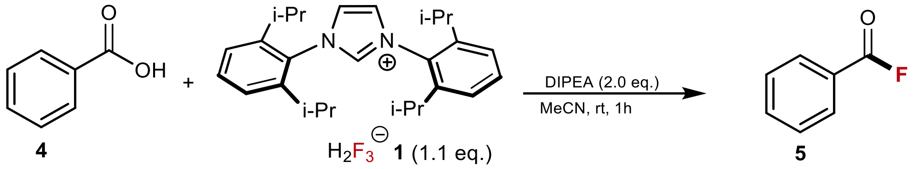


| Entry | Solvent | Base (eq.) | Conditions (T/t) | Reagent (eq.) | Yield (%) |
|---|---|---|---|---|---|
| 1 | PhMe | DBU | rt/4 | 2.0 | 51 |
| 2 | PhMe | DBU | rt/on | 2.0 | 63 |
| 3 | DME | DBU | rt/3 | 2.0 | 92 |
| 4 | DME | DBU | rt/on | 2.0 | 92 |
| 5 | DME | DBU | rt/3 | 1.3 | 63 |
| 6 | MeCN | DIPEA | 140 °C/on | 2.0 | 76 |
| 7 | MeCN | DIPEA | rt/1 h | 1.1 | 98 |

on = overnight

**Table S- 3:** Optimization of reaction conditions for fluorination of phosphates and phosphinates with reagent **1**

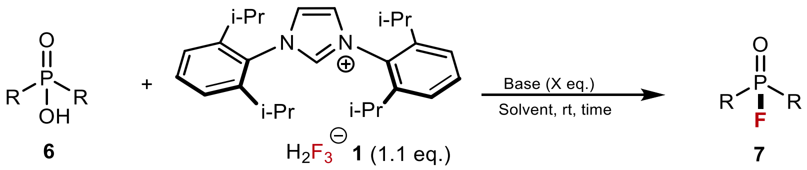


| Entry | Solvent | Base (eq.) | Conditions (T/t) | Reagent (eq.) | Yield (%) |
|---|---|---|---|---|---|
| 1 | MeCN | DIPEA | rt/24 | 1.0 | 66 |
| 2 | MeCN | DIPEA | rt/24 | 2.0 | 89 |
| 3 | PhMe | DIPEA | rt/24 | 1.0 | 13 |
| 4 | PhMe | DIPEA | rt/24 | 2.0 | 80 |
| 5 | MeCN | BTMG | rt/1 | 2.0 | 99 |

## Mechanistic study experiments

In the first step of the reaction benzyl chloride **I** is formed. This step is fast and most of the starting material is consumed within minutes of the addition of the base. The second step of the reaction involves $S_N2$ reaction of the benzyl chloride to yield benzyl fluoride. This step is considerably slower and requires elevated temperatures (Table S- 4).

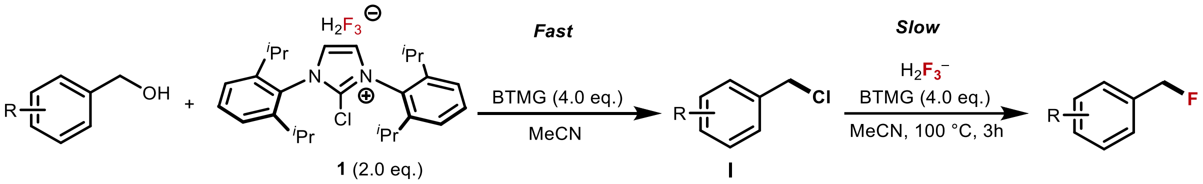

**Table S- 4:** Reaction conditions for determination of reaction steps

| | | | | Product distribution | |
|---|---|---|---|---|---|
| Entry | Substrate | Temperature | Time | $PhCH_2$**Cl** | $PhCH_2$**F** |
| 1 | $PhCH_2$**OH** | rt | 5 min | 100% | 0% |
| 2 | $PhCH_2$**OH** | rt | 24 h | 90% | 10% |
| 3 | $PhCH_2$**Cl** | rt | 24 | 92% | 8 |
| 4 | $PhCH_2$**Cl** | 100 °C | 3 | 33% | 67% |

Benzyl chloride **I**, the product of the first step of the reaction was identified with $^1$H NMR. To show that **I** is later converted to its corresponding fluoride we run the reaction with benzyl chloride instead of benzyl alcohol (Table S-4, entry 3 and 4).

## Hammet correlation experimental details

### Benzyl alcohols

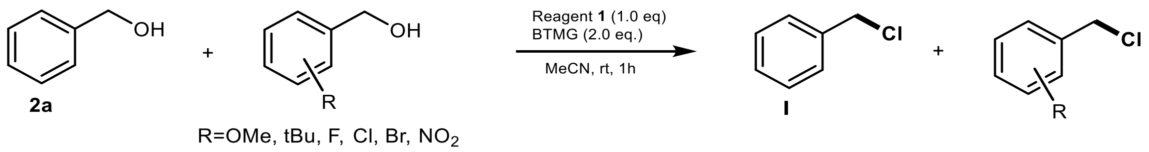


Benyzl alcohol **2a** (0.1 mmol) and substituted benzyl alcohol **2b, 2c, 2d, 2h, 2i, 2j, 2m, 2n** (1.0 eq., 0.1 mmol) were dissolved in dry MeCN (1 mL, 0.1 M) in a glass vial. Reagent **1** (1.0 eq., 48 mg, 0.1 mmol) was added, and the solution was stirred for 1 min until full dissolution. BTMG (2.0 eq., 0.2 mmol, 34.3 mg) was added in one portion, the vial was sealed with a Teflon lined stopper and stirred at room temperature for 1 h. After that 10 µL was transferred to a dry NMR tube and 500 µL of $CDCl_3$ was added. The ratio $X_X/X_H$ between chlorinated products was determined based on the integration ratios of the singlet signal ($-CH_2Cl$). Plot of log($X_X/X_H$) values against corresponding $\sigma$ values was plotted. A line ($R^2$ = 0.967) with $\rho = -0.51$ was obtained.

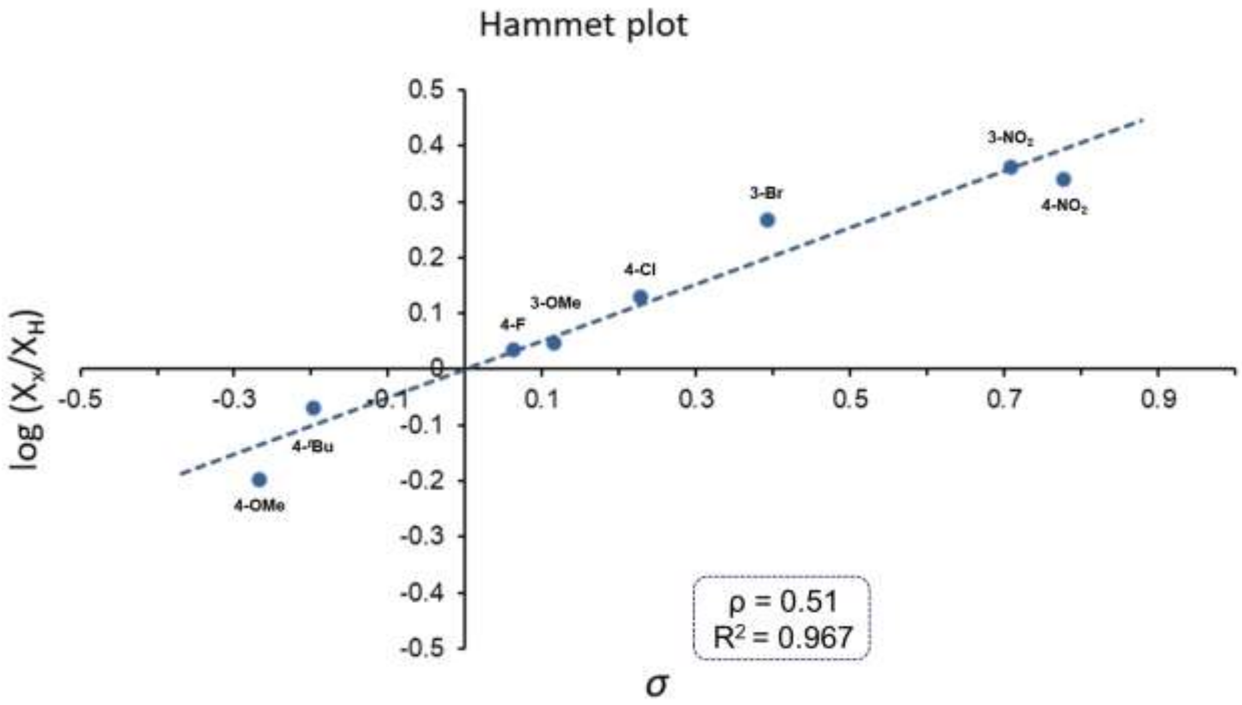


**Graph S- 1:** Hammet plot for substituted benzyl alcohols

**Benzoic acids**

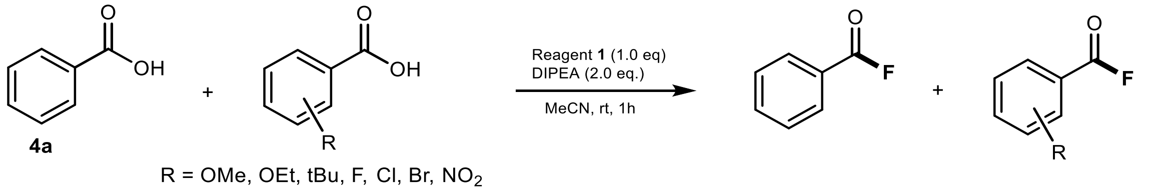


Benzoic acid **4a** (0.1 mmol) and one of the substituted benzoic acids **4c, 4d, 4e, 4f, 4g, 4i, 4j, 4k** (1 eq., 0.1 mmol) were dissolved in dry MeCN (1 mL, 0.1 M) in a glass vial. Reagent **1** (1 eq., 48 mg, 0.1 mmol) was added, and the solution was stirred for 1 min until full dissolution. DIPEA (2 eq., 0.2 mmol, 34.3 mg) was added in one portion, the vial was sealed with a Teflon lined stopper and stirred at room temperature for 0.5 h. After that 10 µL was transferred to a dry NMR tube and 500 µL of $CDCl_3$ was added. The ratio $X_X/X_H$ between fluorinated products was determined based on the integration ratios of the aromatic signals. Plot of log($X_X/X_H$) values against corresponding $\sigma$ values was plotted. A line ($R^2$ = 0.945) with $\rho$ = −2.55 was obtained.

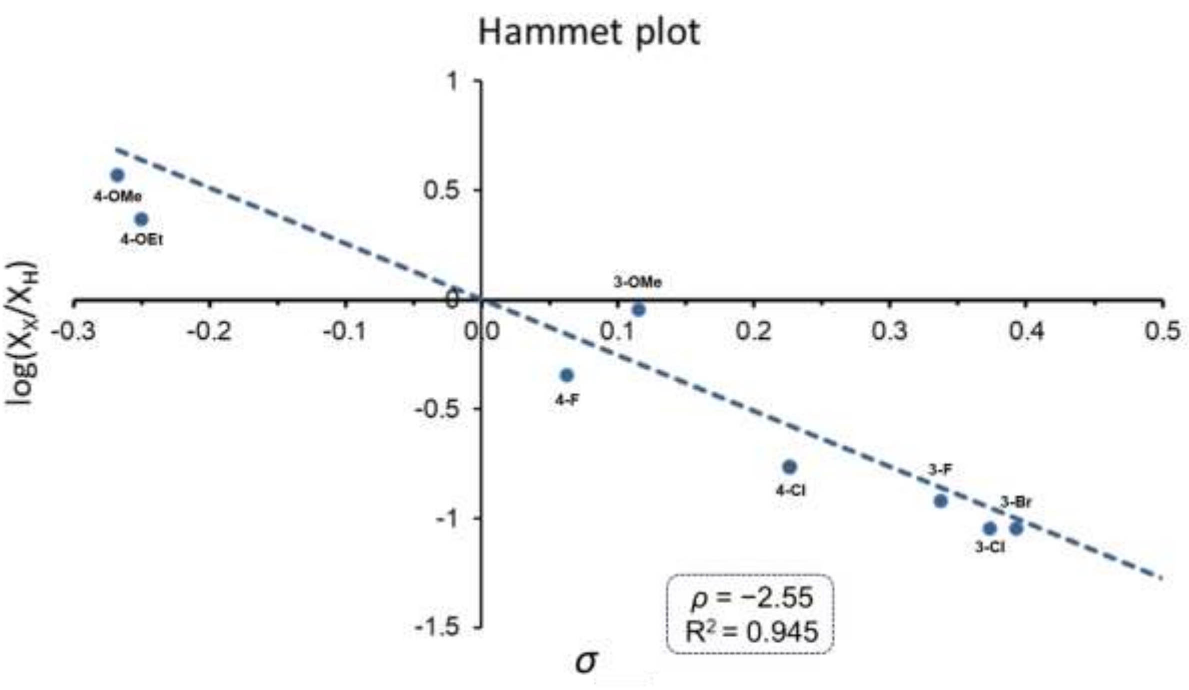


**Figure S- 2**: Hammet plot for substituted benzoic acids

**Phosphinic acids**

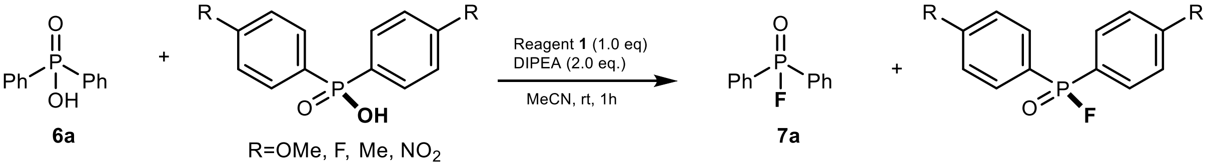


Diphenylphosphinic acid **6a** (0.1 mmol) and substituted diphenylphosphinic acids **6b, 6c, 6d, 6e** (1.0 eq., 0.1 mmol) were dissolved in dry MeCN (1 mL, 0.1 M) in a glass vial. Reagent **1** (1.0 eq., 48 mg, 0.1 mmol) was added, and the solution was stirred for 1 min until full dissolution. DIPEA (2.0 eq., 0.2 mmol, 25.8 mg) was added in one portion, the vial was sealed with a Teflon lined stopper and stirred at room temperature for 1 h. After that 10 µL was transferred to a dry NMR tube and 500 µL of $CDCl_3$ was added. The ratio $X_X/X_H$ between fluorinated products was determined with $^{19}F$ NMR. Plot of $\log(X_X/X_H)$ values against corresponding $\sigma$ values was plotted. A line ($R^2$ = 0.989) with $\rho$ = –2.0 was obtained.

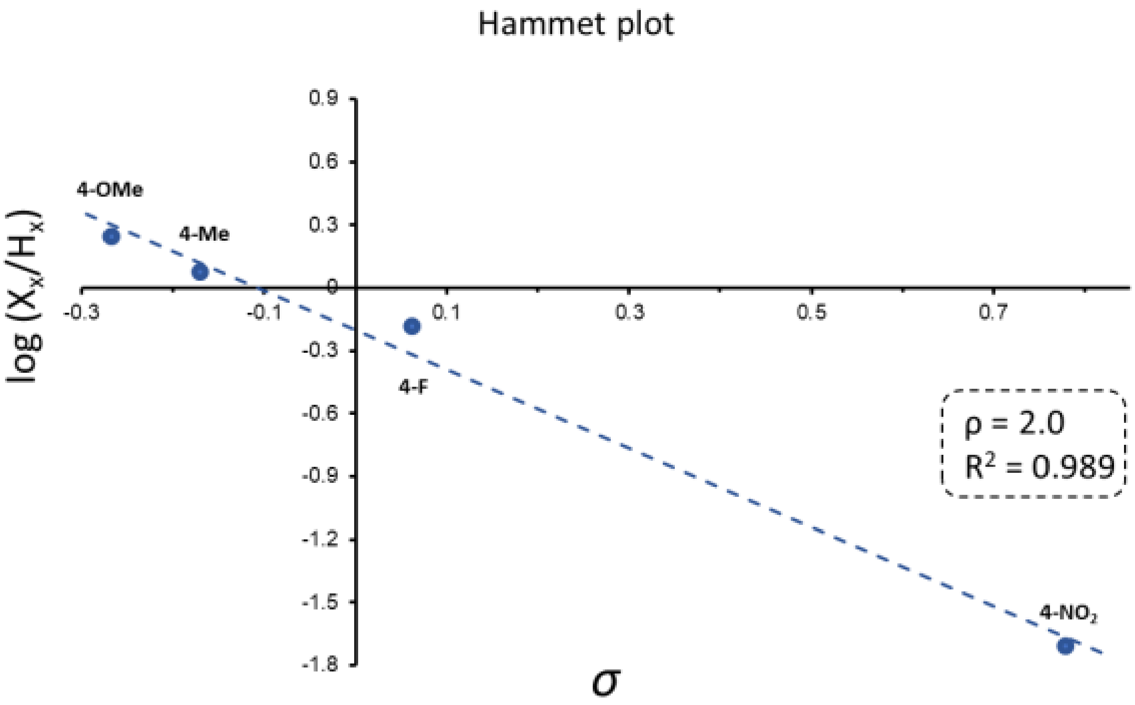


**Graph S- 3:** Hammet plot for substituted diphenylphospinic acids

# Spectroscopic characterization

## Spectroscopic data for benzyl fluorides

Example of NMR yield determination and purity of compounds. NMR yield was determined with naphthalene as an internal standard. Wight amount of standard was added prior to all the reagents and its integral calculated.

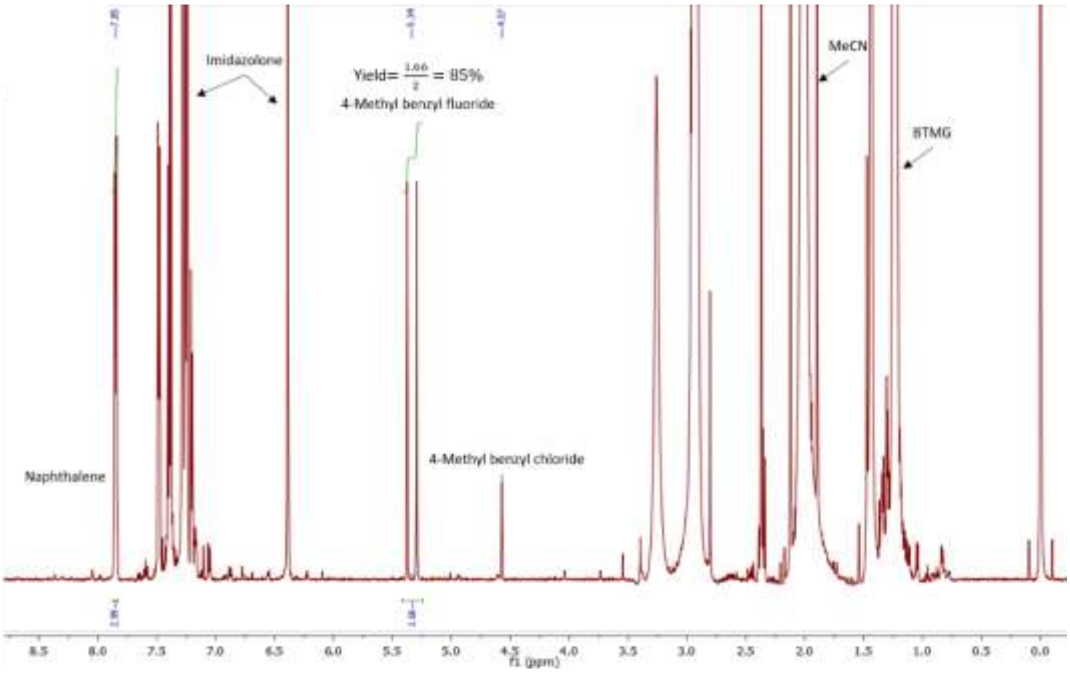


**Figure S- 1:** $^1$H NMR (600 MHz, $CDCl_3$) of the reaction mixture with compound **3l**.

Benzyl fluoride (**3a**)

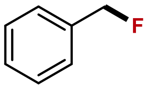

Prepared according to general procedure A for benzyl alcohols. Yield was determined with $^1$H NMR with naphthalene as an internal standard (6.39 mg, 58%). $^1$H NMR (600 MHz, $CDCl_3$, 25 °C): δ 7.42–7.37 (m, 5H), 5.39 (d, *J* = 47.8 Hz, 2H). $^{19}$F NMR (565 MHz, $CDCl_3$, 25 °C): δ –206.6 (t, *J* = 47.7 Hz). Spectroscopic data matched those previously reported in literature.[3]

4–*tert*–butylbenzyl fluoride (**3b**)

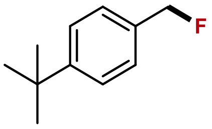

Prepared according to general procedure A for benzyl alcohols. Yield was determined with $^1$H NMR with naphthalene as an internal standard (10.47 mg, 63%). $^1$H NMR (600 MHz, $CDCl_3$, 25 °C): δ 7.45–7.38 (m, 4H), 5.35 (d, *J* = 48.2 Hz, 2H), 1.33 (s, 9H). $^{19}$F NMR (565 MHz, $CDCl_3$, 25 °C): δ –204.2 (t, *J* = 48.7 Hz). Spectroscopic data matched those previously reported in literature.[4]

4–nitrobenzyl fluoride (**3c**)

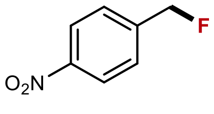

Prepared according to general procedure A for benzyl alcohols. Yield was determined with $^1$H NMR with naphthalene as an internal standard (5.27 mg, 34%). $^1$H NMR (600 MHz, $CDCl_3$, 25 °C): δ 8.26 (d,

2H), 7.54 (d, $J$ = 8.5 Hz, 2H) 5.51 (d, $J$ = 46.8 Hz, 2H). $^{19}$F NMR (565 MHz, $CDCl_3$, 25 °C): δ –215.7 (t, $J$ =46.8 Hz). Spectroscopic data matched those previously reported in literature.[5, 6]

4–methoxybenzyl fluoride (**3d**)

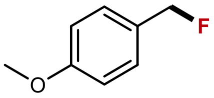


Prepared according to general procedure A for benzyl alcohols. Yield was determined with $^1$H NMR with naphthalene as an internal standard (9.95 mg, 71%). $^1$H NMR (600 MHz, $CDCl_3$, 25 °C): δ 7.42–7.36 (m, 2H), 7.33 (d, $J$ = 8.7 Hz, 2H), 5.30 (d, $J$ = 48.7 Hz, 2H), 3.83 (s, 3H). $^{19}$F NMR (565 MHz, $CDCl_3$, 25 °C): –199.1 (t, $J$ = 48.7 Hz). Spectroscopic data matched those previously reported in literature.[7]

2–fluorobenzyl fluoride (**3e**)

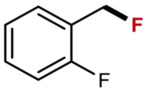


Prepared according to general procedure A for benzyl alcohols. Yield was determined with $^1$H NMR with naphthalene as an internal standard (9.99 mg, 78%). $^1$H NMR (600 MHz, $CDCl_3$, 25 °C): δ 7.45–7.35 (m, 4H), 5.47 (d, $J$ = 48.2 Hz, 2H). $^{19}$F NMR (565 MHz, $CDCl_3$, 25 °C): δ –118.7 (s, 1F), –212.5 (t, $J$ = 47.6 Hz, 1F). Spectroscopic data matched those previously reported in literature.[8]

2–bromobenzyl fluoride (**3f**)

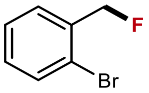


Prepared according to general procedure A for benzyl alcohols. Yield was determined with $^1$H NMR with naphthalene as an internal standard (16.26 mg, 86%). $^1$H NMR (600 MHz, $CDCl_3$, 25 °C): δ 7.57 (d, $J$ = 8.0 Hz, 1H), 7.48 (m, 1H), 7.38 (d, $J$ = 7.6 Hz,1H), 7.23 (d, $J$ = 7.8 Hz 1H), 5.47 (d, $J$ = 47.2 Hz, 2H). $^{19}$F NMR (565 MHz, $CDCl_3$, 25 °C): δ –216.3 (t, $J$ = 47.2 Hz). Spectroscopic data matched those previously reported in literature.[9]

2–iodobenzyl fluoride (**3g**)

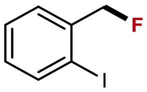


Prepared according to general procedure A for benzyl alcohols. Yield was determined with $^1$H NMR with naphthalene as an internal standard (19.59 mg, 83%). $^1$H NMR (600 MHz, $CDCl_3$, 25 °C): δ 7.85 (m, 1H), 7.45–7.36 (m, 2H), 7.06 (t, $J$ = 7.6 Hz), 5.39 (d, $J$ = 47.0 Hz, 2H). $^{19}$F NMR (565 MHz, $CDCl_3$, 25 °C): δ –213.4 (t, $J$ = 48.7 Hz). Spectroscopic data matched those previously reported in literature.[10]

4–chlorobenzyl fluoride (**3h**)

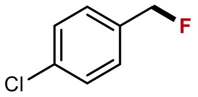

Prepared according to general procedure A for benzyl alcohols. Yield was determined with $^{1}H$ NMR with naphthalene as an internal standard (11.71 mg, 81%). $^{1}H$ NMR (600 MHz, $CDCl_3$, 25 °C): δ 7.38 (d, $J$ = 7.6 Hz, 2H), 7.33 (m, 2H), 5.35 (d, $J$ = 48.2 Hz, 2H). $^{19}F$ NMR (565 MHz, $CDCl_3$, 25 ° C): δ –207.4 (t, $J$ = 48.7 Hz). Spectroscopic data matched those previously reported in literature.[9]

3–bromobenzyl fluoride (**3i**)

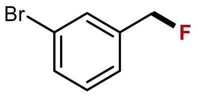

Prepared according to general procedure A for benzyl alcohols. Yield was determined with $^{1}H$ NMR with naphthalene as an internal standard (16.07 mg, 85%). $^{1}H$ NMR (600 MHz, $CDCl_3$, 25 °C): δ 7.45–7.55 (m, 2H), 7.22–7.31 (m, 2H), 5.35 (d, $J$ = 48.2 Hz, 2H). $^{19}F$ NMR (565 MHz, $CDCl_3$, 25 °C): δ –209.8 (t, $J$ = 48.7 Hz). Spectroscopic data matched those previously reported in literature.[10]

4–fluorobenzyl fluoride (**3j**)

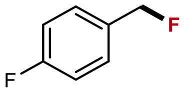

Prepared according to general procedure A for benzyl alcohols. Yield was determined with $^{1}H$ NMR with naphthalene as an internal standard (9.74 mg, 76%). $^{1}H$ NMR (600 MHz, $CDCl_3$, 25 °C): δ 7.43–7.35 (m, 2H), 7.09 (td, $J$ = 8.6, 1.1 Hz, 2H), 5.35 (d, $J$ = 48.2 Hz, 2H). $^{19}F$ NMR (565 MHz, $CDCl_3$, 25 °C): δ –112.9 (m), –203.9 (t, $J$ = 48.2 Hz). Spectroscopic data matched those previously reported in literature.[11]

4–trifluoromethylthiobenzyl fluoride (**3k**)

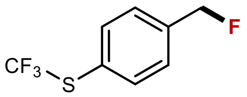

Prepared according to general procedure A for benzyl alcohols. Yield was determined with $^{1}H$ NMR with naphthalene as an internal standard (17.24 mg, 82%). The product was isolated by the following procedure: The solvent was evaporated, and the residue was dissolved in a mixture of aqueous HCl 0.5 M (15 mL) and dichloromethane (15 mL). After phase separation, the solvent from organic phase was evaporated and the product was purified by silica gel column flash chromatography using hexane and dichloromethane (3:2) as eluent (mixture of 4-trifluoromethylthiobenzyl fluoride **3k** and 4-trifluoromethylthiobenzyl chloride 1 : 1.7, clear liquid). $^{1}H$ NMR (600 MHz, $CDCl_3$, 25 °C): δ 7.68 (d, $J$ = 7.6 Hz, 2H), 7.42 (d, $J$ = 7.4 Hz, 2H), 5.43 (d, $J$ = 47.2 Hz, 2H). $^{19}F$ NMR (565 MHz, $CDCl_3$, 25 °C): δ –212.4 (t, $J$ = 47.2 Hz, 1F), –42,7 (s, 3F). $^{13}C\{^{1}H\}$ NMR (151 MHz, $CDCl_3$, 25 °C) δ 139.3 (d, $J$ = 17.6 Hz), 136.5, 129.4 (q, $J$ = 308 Hz), 127.8 (d, $J$ = 6.5 Hz), 124.7 – 124.6 (m), 83.5 (d, $J$ = 168.9 Hz). HRMS (ESI-TOF): m/z $M^+$ calculated for $C_8H_6SF_4$: 210.0126; found: 210.0135.

4–methylbenzyl fluoride (**3l**)

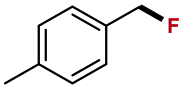


Prepared according to general procedure A for benzyl alcohols. Yield was determined with $^1$H NMR with naphthalene as an internal standard (16.07 mg, 85%). $^1$H NMR (600 MHz, $CDCl_3$, 25 °C): δ 7.39 (d, *J* = 7.6 Hz, 2H), 7.25 (d, *J* = 7.7 Hz, 2H), 5.33 (d, *J* = 48.2 Hz, 2H), 2.37 (d, *J* = 3.1 Hz, 3H). $^{19}$F NMR (565 MHz, $CDCl_3$, 25 °C): −204.6 (t, *J* = 48.2 Hz). Spectroscopic data matched those previously reported in literature.[7, 12]

3–nitrobenzyl fluoride (**3m**)

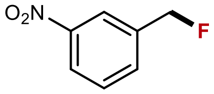


Prepared according to general procedure A for benzyl alcohols. Yield was determined with $^1$H NMR with naphthalene as an internal standard (12.88 mg, 83%). $^1$H NMR (600 MHz, $CDCl_3$, 25 °C): δ 8.29 – 8.19 (m, 2H), 7.71 (d, *J* = 7.7 Hz, 1H), 7.60 (t, *J* = 7.9 Hz, 1H), 5.50 (d, *J* = 47.0 Hz, 2H). $^{19}$F NMR (565 MHz, $CDCl_3$, 25 °C): δ –212.4 (t, *J* = 47.0 Hz). Spectroscopic data matched those previously reported in literature.[13]

2–methylbenzyl fluoride (**3n**)

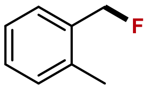


Prepared according to general procedure A for benzyl alcohols. Yield was determined with $^1$H NMR with naphthalene as an internal standard (9.06 mg, 73%). $^1$H NMR (600 MHz, $CDCl_3$, 25 °C): δ 7.39 (t, *J* = 7.8 Hz, 2H), 7.26 – 7.19 (m, 2H), 5.42 (d, *J* = 47.9 Hz, 2H), 2.38 (d, *J* = 1.7 Hz, 3H). $^{19}$F NMR (565 MHz, $CDCl_3$, 25 °C): δ –163.0 (m). Spectroscopic data matched those previously reported in literature.[14]

2–(fluoromethyl)naphthalene (**3o**)

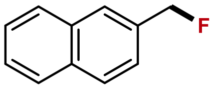


Prepared according to general procedure A for benzyl alcohols. Yield was determined with $^1$H NMR with naphthalene as an internal standard (8.49 mg, 53%). $^1$H NMR (600 MHz, $CDCl_3$, 25 °C): δ 7.90 – 7.83 (m, 4H), 7.62 – 7.56 (m, 3H), 5.55 (d, *J* = 47.8 Hz, 2H). $^{19}$F NMR (565 MHz, $CDCl_3$, 25 °C): δ –206.7 (t, *J* = 47.8 Hz). Spectroscopic data matched those previously reported in literature.[15, 16]

1–(2–fluoroethyl)–2–methyl–5–nitro–1*H*–imidazole (**3p**)

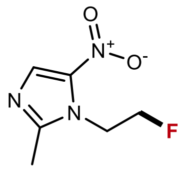

Prepared according to general procedure A for benzyl alcohols. Yield was determined with $^1$H NMR with naphthalene as an internal standard (11.95 mg, 69%). $^1$H NMR (600 MHz, $CDCl_3$, 25 °C): δ 7.98 (s, 1H), 4.78 (dt, $J$ = 46.9, 4.4 Hz, 2H), 4.64 (dt, $J$ = 25.9, 4.4 Hz, 2H), 2.46 (s, 3H). $^{19}$F NMR (565 MHz, $CDCl_3$, 25 °C): δ –244.2 (tt, $J_1$ = 47.2, $J_2$ = 25.9 Hz). Spectroscopic data matched those previously reported in literature.[17]

(2*R*,3*R*,4*S*,5*R*,6*S*)-3,4,5-tris(benzyloxy)-2-((benzyloxy)methyl)-6-fluorotetrahydro-2*H*-pyran (**3r**)

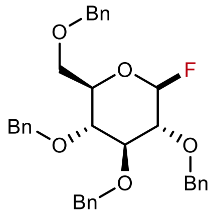

Prepared according to general procedure A for benzyl alcohols. Yield was determined with $^1$H NMR with naphthalene as an internal standard (27.13 mg, 50%, 30:70 $\alpha$:$\beta$). $^1$H NMR (600 MHz, $CDCl_3$, 25 °C): δ 7.36 – 7.25 (m, 18H), 7.16–7.12 (m, 2H), 5.25 (dd, $J_1$ = 52.9 Hz, $J_2$ = 6.8 Hz, 1H), 4.99 – 4.44 (m, 8H), 3.88 – 3.85 (m, 4H), 3.62 – 3.56 (m, 2H). $^{19}$F NMR (565 MHz, $CDCl_3$, 25 °C): δ –138.1 (dd, $J_1$ = 52.8, $J_2$ = 12.1 Hz, $\beta$), –149.6 (dd, $J_1$ = 52.9, $J_2$ = 25.7 Hz, $\alpha$). Spectroscopic data matched those previously reported in literature.[6, 18]

## Spectroscopic data for benzoyl fluorides

Example of NMR yield determination and purity of compounds. NMR yield was determined with naphthalene as an internal standard. Wight amount of standard was added prior to all the reagents and its integral calculated.

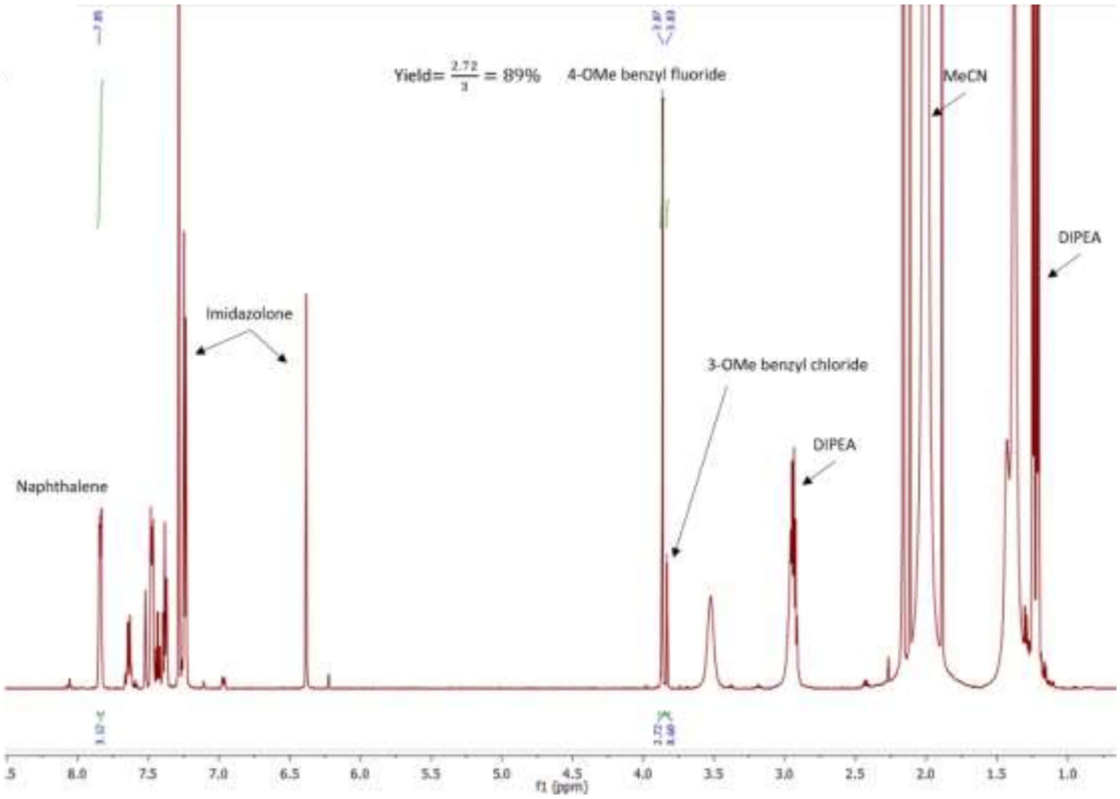


**Figure S- 2:** $^{1}$H NMR (600 MHz, $CDCl_3$) of the reaction mixture with compound **5l**.

Benzoyl fluoride (**5a**)

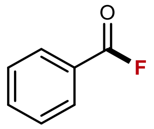


Prepared according to general procedure B for benzoic acids. Yield was determined with $^{1}$H NMR with naphthalene as an internal standard (12.16 mg, 98%). $^{1}$H NMR (600 MHz, $CDCl_3$, 25 °C): δ 8.05 (d, $J$ = 7.2 Hz, 2H), 7.71 (t, $J$ = 7.5 Hz, 1H), 7.56 – 7.52 (m, 2H).$^{19}$F NMR (565 MHz, $CDCl_3$, 25 °C): δ 18.0 (s). Spectroscopic data matched those previously reported in literature.[19]

4-nitrobenzoyl fluoride (**5b**)

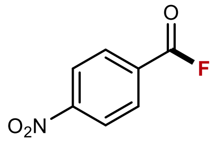


Prepared according to general procedure B for benzoic acids. Yield was determined with $^{1}$H NMR with naphthalene as an internal standard (6.76 mg, 40%). $^{1}$H NMR (600 MHz, $CDCl_3$, 25 °C): δ 8.39 (d, $J$ = 8.4 Hz, 2H), 8.26 (d, $J$ = 8.7 Hz, 2H). $^{19}$F NMR (565 MHz, $CDCl_3$, 25 °C): δ 21.3 (s). Spectroscopic data matched those previously reported in literature.[20]

4-methoxybenzoyl fluoride (**5c**)

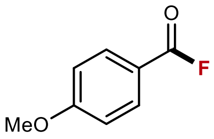


Prepared according to general procedure B for benzoic acids. Yield was determined with $^{1}H$ NMR with naphthalene as an internal standard (12.02 mg, 78%). $^{1}H$ NMR (600 MHz, $CDCl_3$, 25 °C): 8.00 (d, *J* = 8.6 Hz, 2H), 6.99 (d, *J* = 8.5 Hz, 2H), 3.90 (s, 3H). $^{19}F$ NMR (565 MHz, $CDCl_3$, 25 °C): δ 16.0 (s). Spectroscopic data matched those previously reported in literature.[19]

4-chlorobenzoyl fluoride (**5d**)

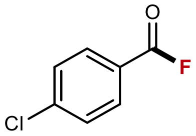


Prepared according to general procedure B for benzoic acids. Yield was determined with $^{1}H$ NMR with naphthalene as an internal standard (14.27 mg, 90%). $^{1}H$ NMR (600 MHz, $CDCl_3$, 25 °C): δ 7.99 (d, *J* = 8.3 Hz, 2H), 7.52 (d, *J* = 8.3 Hz, 2H). $^{19}F$ NMR (565 MHz, $CDCl_3$, 25 °C): δ 18.4 (s). Spectroscopic data matched those previously reported in literature.[20]

4-ethoxybenzoyl fluoride (**5e**)

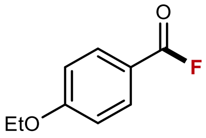


Prepared according to general procedure B for benzoic acids. Yield was determined with $^{1}H$ NMR with naphthalene as an internal standard (15.47 mg, 92%). $^{1}H$ NMR (600 MHz, $CDCl_3$, 25 °C): δ 7.98 (d, *J* = 8.8 Hz, 2H), 6.97 (d, *J* = 8.6 Hz, 2H), 4.13 (q, *J* = 7.0 Hz, 2H), 1.46 (t, *J* = 7.0 Hz, 3H). $^{19}F$ NMR (565 MHz, $CDCl_3$, 25 °C): δ 15.8 (s). Spectroscopic data matched those previously reported in literature.[21]

4–fluorobenzoyl fluoride (**5f**)

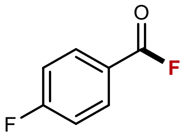


Prepared according to general procedure B for benzoic acids. Yield was determined with $^{1}H$ NMR with naphthalene as an internal standard (10.23 mg, 72%). $^{1}H$ NMR (600 MHz, $CDCl_3$, 25 °C): δ 8.11–8.06 (m, 2H), 7.26–7.17 (m, 2H). $^{19}F$ NMR (565 MHz, $CDCl_3$, 25 °C): δ 18.0 (s), –100.6 (m). Spectroscopic data matched those previously reported in literature.[20]

3-fluorobenzoyl fluoride (**5g**)

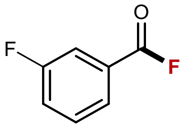

Prepared according to general procedure B for benzoic acids. Yield was determined with $^{1}$H NMR with naphthalene as an internal standard (12.93 mg, 91%). $^{1}$H NMR (600 MHz, $CDCl_3$, 25 °C): δ 7.86 (d, 1H), 7.73 (d, *J* = 8.7 Hz, 1H), 7.53 (q, *J* = 7.7 Hz, 1H), 7.45–7.42 (m, 1H). $^{19}$F NMR (565 MHz, $CDCl_3$, 25 °C): δ 19.3 (s), –110.6 (m). Spectroscopic data matched those previously reported in literature.[19, 22]

3-nitrobenzoyl fluoride (**5h**)

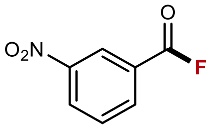

Prepared according to general procedure B for benzoic acids. Yield was determined with $^{1}$H NMR with naphthalene as an internal standard (9.64 mg, 57%). $^{1}$H NMR (600 MHz, $CDCl_3$, 25 °C): δ 8.90 (m, 1H), 8.40 (t, *J* = 7.9 Hz, 1H), 8.24 (m, 1H), 7.79 (t, *J* = 8.0 Hz, 1H). $^{19}$F NMR (565 MHz, $CDCl_3$, 25 °C): δ 20.2 (s). Spectroscopic data matched those previously reported in literature.[23]

3-bromobenzoyl fluoride (**5i**)

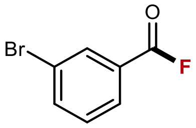

Prepared according to general procedure B for benzoic acids. Yield was determined with $^{1}$H NMR with naphthalene as an internal standard (8.73 mg, 43%). $^{1}$H NMR (600 MHz, $CDCl_3$, 25 °C): 8.19 (t, 1H), 7.99 (d, *J* = 7.7 Hz, 1H), 7.84 (m, 1H), 7.44 (m, 1H). $^{19}$F NMR (565 MHz, $CDCl_3$, 25 °C): δ 19.1 (s). Spectroscopic data matched those previously reported in literature.[20]

3-chlorobenzoyl fluoride (**5j**)

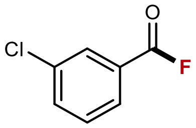

Prepared according to general procedure B for benzoic acids. Yield was determined with $^{1}$H NMR with naphthalene as an internal standard (12.53 mg, 79%). $^{1}$H NMR (600 MHz, $CDCl_3$, 25 °C): δ 8.03 (m, 1H), 7.95 (d, *J* = 7.8 Hz, 1H), 7.69 (m, 1H), 7.49 (m, 1H). $^{19}$F NMR (565 MHz, $CDCl_3$, 25 °C): δ 19.1 (s). Spectroscopic data matched those previously reported in literature.[24]

3-methoxybenzoyl fluoride (**5k**)

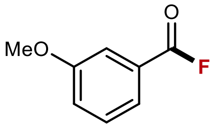


Prepared according to general procedure B for benzoic acids. Yield was determined with $^{1}$H NMR with naphthalene as an internal standard (13.41 mg, 87%). $^{1}$H NMR (600 MHz, $CDCl_3$, 25 °C): δ 7.65 (d, *J* = 7.6 Hz, 1H), 7.53 (d, *J* = 2.4 Hz, 1H), 7.43 (m, 1H), 7.30 (m, 1H). $^{19}$F NMR (565 MHz, $CDCl_3$, 25 °C): δ 18.6 (s). Spectroscopic data matched those previously reported in literature.[22]

2-iodobenzoyl fluoride (**5l**)

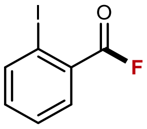


Prepared according to general procedure B for benzoic acids. Yield was determined with $^{1}$H NMR with naphthalene as an internal standard (20.75 mg, 83%). $^{1}$H NMR (600 MHz, $CDCl_3$, 25 °C): δ 8.14 (d, *J* = 8.0 Hz, 1H), 8.03 (dd, $J_1$ = 7.8, $J_2$ = 1.7 Hz, 1H), 7.50 (m, 1H), 7.31 (m, 1H). $^{19}$F NMR (565 MHz, $CDCl_3$, 25 °C): δ 28.7 (s). Spectroscopic data matched those previously reported in literature.[25]

2-bromobenzoyl fluoride (**5m**)

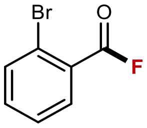


Prepared according to general procedure B for benzoic acids. Yield was determined with $^{1}$H NMR with naphthalene as an internal standard (17.26 mg, 85%). $^{1}$H NMR (600 MHz, $CDCl_3$, 25 °C): δ 8.03 (dd, $J_1$ = 7.5 Hz, $J_2$ = 2.1 Hz, 1H), 7.79 (m, 1H), 7.50 (m, 2H). $^{19}$F NMR (565 MHz, $CDCl_3$, 25 °C): δ 31.5 (s). Spectroscopic data matched those previously reported in literature.[20]

2-fluorobenzoyl fluoride (**5n**)

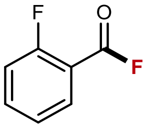


Prepared according to general procedure B for benzoic acids. Yield was determined with $^{1}$H NMR with naphthalene as an internal standard (12.08 mg, 85%). $^{1}$H NMR (600 MHz, $CDCl_3$, 25 °C): δ 7.99 (t, *J* = 7.5 Hz, 1H), 7.70 (q, *J* = 7.1 Hz, 1H), 7.34–7.21 (m, 2H). $^{19}$F NMR (565 MHz, $CDCl_3$, 25 °C): δ 31.6 (s), –106.21 (m). Spectroscopic data matched those previously reported in literature.[20]

1-naphthoyl fluoride (**5o**)

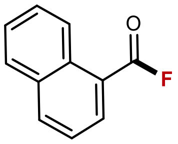


Prepared according to general procedure B for benzoic acids. Yield was determined with $^1$H NMR with naphthalene as an internal standard (14.80 mg, 85%). $^1$H NMR (600 MHz, $CDCl_3$, 25 °C): δ 9.02 (d, $J$ = 8.7 Hz, 1H), 8.37 (dd, $J_1$ = 7.3, $J_2$ = 1.2 Hz, 1H), 8.20 (d, $J$ = 8.2 Hz, 1H), 7.95 (d, $J$ = 8.2 Hz, 1H), 7.73 (ddd, $J_1$ = 8.5, $J_2$ = 6.8, $J_3$ = 1.3 Hz, 1H), 7.64 – 7.61 (m, 1H), 7.60 – 7.57 (m, 1H). $^{19}$F NMR (565 MHz, $CDCl_3$, 25 °C): δ 29.9 (s). Spectroscopic data matched those previously reported in literature.[24]

2,4-dichlorobenzoyl fluoride (**5q**)

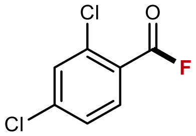


Prepared according to general procedure B for benzoic acids. Yield was determined with $^1$H NMR with naphthalene as an internal standard (14.86 mg, 77%). $^1$H NMR (600 MHz, $CDCl_3$, 25 °C): δ 7.97 (d, $J$ = 8.5 Hz, 1H), 7.60 (m, 1H), 7.41 (m, 1H). $^{19}$F NMR (565 MHz, $CDCl_3$, 25 °C): δ 31.9 (s). Spectroscopic data matched those previously reported in literature.[26]

2,2-diphenylacetyl fluoride (**5r**)

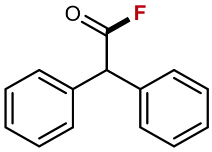


Prepared according to general procedure B for benzoic acids. Yield was determined with $^1$H NMR with naphthalene as an internal standard (15.21 mg, 71%). $^1$H NMR (600 MHz, $CDCl_3$, 25 °C): δ 7.40–7.29 (m, 10H), 5.16 (s, 1H). $^{19}$F NMR (565 MHz, $CDCl_3$, 25 °C): δ 44.9 (s). Spectroscopic data matched those previously reported in literature.[27]

2-phenylpropanoyl fluoride (**5s**)

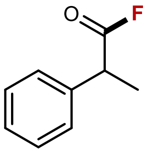


Prepared according to general procedure B for benzoic acids. Yield was determined with $^1$H NMR with naphthalene as an internal standard (13.39 mg, 88%). $^1$H NMR (600 MHz, $CDCl_3$, 25 °C): δ 7.35–7.29 (m, 5H), 3.87 (q, $J$ = 7.4 Hz, 1H), 1.51 (d, $J$ = 7.2 Hz, 3H). $^{19}$F NMR (565 MHz, $CDCl_3$, 25 °C): δ 39.6 (s). Spectroscopic data matched those previously reported in literature.[28]

3,5-dimethoxybenzoyl fluoride (**5t**)

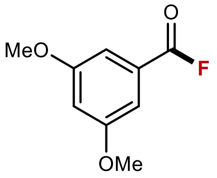


Prepared according to general procedure B for benzoic acids. Yield was determined with $^1$H NMR with naphthalene as an internal standard (16.76 mg, 91%). $^1$H NMR (600 MHz, $CDCl_3$, 25 °C): δ 7.17 (d, *J* = 2.3 Hz, 2H), 6.76 (t, *J* = 2.3 Hz, 1H), 3.51 (s, 6H). $^{19}$F NMR (565 MHz, $CDCl_3$, 25 °C): δ 18.8 (s). Spectroscopic data matched those previously reported in literature.[29]

## Spectroscopic data for amino acid fluorides and biologically active compounds

*tert*-butyl (*S*)-(1-fluoro-1-oxopropan-2-yl)carbamate (**5u**)

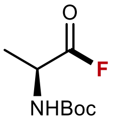


Prepared according to general procedure B for benzoic acids. Yield was determined with $^1$H NMR with naphthalene as an internal standard (17.59 mg, 92%). $^1$H NMR (600 MHz, $CDCl_3$, 25 °C): δ 4.95 (s, 1H), 4.47 (s, 1H), 1.50 (d, *J* = 7.3 Hz, 3H), 1.46 (s, 9H). $^{19}$F NMR (565 MHz, $CDCl_3$, 25 °C): δ 27.8 (s). Spectroscopic data matched those previously reported in literature.[30]

*tert*-butyl (*S*)-(1-fluoro-1-oxo-3-phenylpropan-2-yl)carbamate (**5v**)

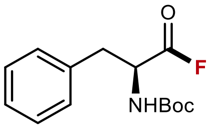


Prepared according to general procedure B for benzoic acids. Yield was determined with $^1$H NMR with naphthalene as an internal standard (26.46 mg, 99%). $^1$H NMR (600 MHz, $CDCl_3$, 25 °C): δ 7.30–7.10 (m, 5H), 4.86 (m, 1H), 4.74 (m, 1H), 3.21–3.11 (m, 2H), 1.42 (s, 9H). $^{19}$F NMR (565 MHz, $CDCl_3$, 25 °C): δ 30.8 (s). Spectroscopic data matched those previously reported in literature.[31]

*tert*-butyl ((2*S*,3*S*)-1-fluoro-3-methyl-1-oxopentan-2-yl)carbamate (**5w**)

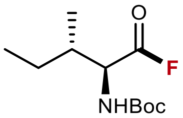


Prepared according to general procedure B for benzoic acids. Yield was determined with $^1$H NMR with naphthalene as an internal standard (23.33 mg, >99%). $^1$H NMR (600 MHz, $CDCl_3$, 25 °C): δ 4.94 (m, 1H), 4.43 (m, 1H), 1.52–1.48 (m, 10H), 1.24 (m, 1H), 1.03 (m, 3H), 0.96 (t, *J* = 7.4 Hz, 3H). $^{19}$F NMR (565 MHz, $CDCl_3$, 25 °C): δ 35.1 (s). Spectroscopic data matched those previously reported in literature.[32]

*tert*-butyl (*S*)-(1-fluoro-3-(1H-indol-3-yl)-1-oxopropan-2-yl)carbamate (**5x**)

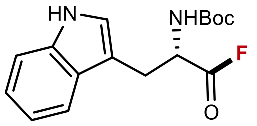


Prepared according to general procedure B for benzoic acids. Yield was determined with $^{1}$H NMR with naphthalene as an internal standard (30.63 mg, >99%). $^{1}$H NMR (600 MHz, $CDCl_3$, 25 °C): δ 7.64 (t, $J$ = 7.7 Hz, 1H), 7.57 (d, $J$ = 8.1 Hz, 1H), 7.21 (dd, $J_1$ = 8.3, $J_2$ = 7.1 Hz, 1H), 7.14 (t, $J$ = 7.5 Hz, 1H), 7.09 (t, $J$ = 3.0 Hz, 1H), 5.03 – 4.78 (m, 2H), 3.67 (m, 1H), 1.43 (s, 9H). $^{19}$F NMR (565 MHz, $CDCl_3$, 25 °C): δ 35.1 (s). Spectroscopic data matched those previously reported in literature.[33]

*tert*-butyl (2-fluoro-2-oxoethyl)carbamate (**5y**)

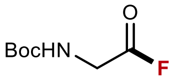


Prepared according to general procedure B for benzoic acids. Yield was determined with $^{1}$H NMR with naphthalene as an internal standard (16.65 mg, 94%). $^{1}$H NMR (600 MHz, $CDCl_3$, 25 °C): δ 4.11 (2H, t, $J$ = 5.5 Hz), 1.47 (9H, s). $^{19}$F NMR (565 MHz, $CDCl_3$, 25 °C): δ 31.8 (s). Spectroscopic data matched those previously reported in literature.[31]

## Bioactive derivatives

(*S*)-2-(6-methoxynaphthalen-2-yl)propanoyl fluoride (**5aa**)

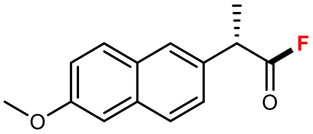


Prepared according to general procedure B for benzoic acids. Yield was determined with $^{1}$H NMR with naphthalene as an internal standard (18.58 mg, 80%). $^{1}$H NMR (600 MHz, $CDCl_3$, 25 °C): δ 7.77–7.69 (m, 3H), 7.39 (m, 1H), 7.17 (dd, $J_1$ = 8.9 Hz, $J_2$ = 2.6 Hz, 1H), 7.13 (d, $J$ = 2.6, 1H), 4.01 (q, $J$ = 7.3 Hz, 1H), 3.91 (s, 3H), 1.68 (d, $J$ = 7.2, 3H). $^{19}$F NMR (565 MHz, $CDCl_3$, 25 °C): δ 39.6 (s). Spectroscopic data matched those previously reported in literature.[34]

2-(fluorocarbonyl)phenyl acetate (**5ab**)

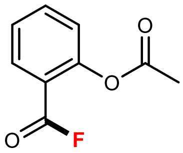


Prepared according to general procedure B for benzoic acids. Yield was determined with $^{1}$H NMR with naphthalene as an internal standard (11.66 mg, 64%). $^{1}$H NMR (600 MHz, $CDCl_3$, 25 °C): δ 8.04 (dd, $J_1$ = 7.9, $J_2$ = 1.7 Hz, 1H), 7.73 (td, $J$ = 7.8, 1.7 Hz, 1H), 7.40 (m, 1H), 7.21 (d, $J$ = 8.2 Hz, 1H), 2.38 (s, 3H). $^{19}$F NMR (565 MHz, $CDCl_3$, 25 °C): δ 28.5 (s). Spectroscopic data matched those previously reported in literature.[35]

2-(4-isobutylphenyl)propanoyl fluoride (**5ac**)

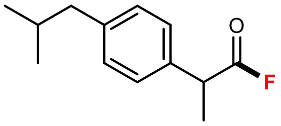


Prepared according to general procedure B for benzoic acids. Yield was determined with $^{1}H$ NMR with naphthalene as an internal standard (16.66 mg, 80%). $^{1}H$ NMR (600 MHz, $CDCl_3$, 25 °C): δ 7.20 (d, 2H), 7.14 (d, *J* = 7.9 Hz, 2H), 3.84 (q, *J* = 7.1 Hz, 1H), 2.46 (d, *J* = 7.2 Hz, 2H), 1.94 – 1.82 (m, 1H), 1.58 (d, *J* = 7.2 Hz, 3H), 0.90 (d, *J* = 6.6 Hz, 6H). $^{19}F$ NMR (565 MHz, $CDCl_3$, 25 °C): δ 39.3 (s). Spectroscopic data matched those previously reported in literature.[19]

nicotinoyl fluoride (**5ad**)

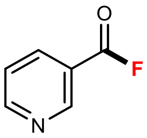


Prepared according to general procedure B for benzoic acids. Yield was determined with $^{1}H$ NMR with naphthalene as an internal standard (10.01 mg, 80%). $^{1}H$ NMR (600 MHz, $CDCl_3$, 25 °C): δ 9.26 (d, *J* = 2.1 Hz, 1H), 8.92 (dd, $J_1$ = 4.9, $J_2$ = 1.7 Hz, 1H), 8.32 (dt, *J* = 7.9, 2.1 Hz, 1H), 7.49 (m, 1H). $^{19}F$ NMR (565 MHz, $CDCl_3$, 25 °C): δ 20.7 (s). Spectroscopic data matched those previously reported in literature.[19]

## Spectroscopic data for fluoro phosphates and phosphinates

Example of NMR yield determination and purity of compounds. NMR yield was determined with naphthalene as an internal standard. Wight amount of standard was added prior to all the reagents and its integral calculated.

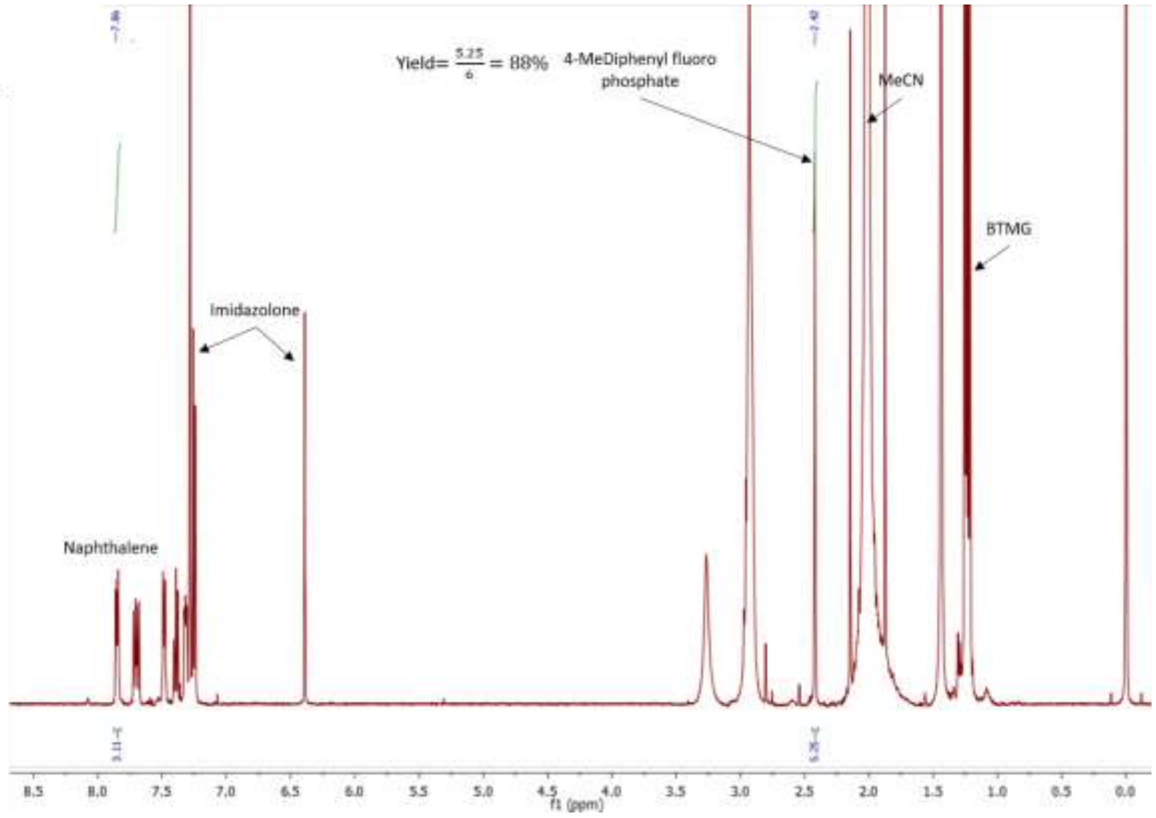


**Figure S- 3:** $^{1}H$ NMR (600 MHz, $CDCl_3$) of the reaction mixture with compound **7b**.

diphenylphosphinic fluoride (**7a**)

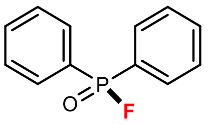


Prepared according to general procedure C for phosphinic acids and hydrogenphosphates. Yield was determined with $^{1}H$ NMR with naphthalene as an internal standard (22.02 mg, >99%). $^{1}H$ NMR (600 MHz, $CDCl_3$, 25 °C): δ 7.81–7.86 (m, 4H), 7.51–7.55 (m, 2H), 7.36–7.42 (m, 4H). $^{19}F$ NMR (565 MHz, $CDCl_3$, 25 °C): δ –75.2 (d, $J$ = 1019 Hz). $^{31}P$ NMR (243 MHz, $CDCl_3$, 25 °C): δ 40.9 (d, $J$ = 1019 Hz). Spectroscopic data matched those previously reported in literature.[36]

di-*p*-tolylphosphinic fluoride (**7b**)

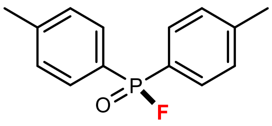


Prepared according to general procedure C for phosphinic acids and hydrogenphosphates. Yield was determined with $^{1}H$ NMR with naphthalene as an internal standard (21.84 mg, 88%). $^{1}H$ NMR (600 MHz, $CDCl_3$, 25 °C): δ 7.70 (m, 4H), 7.32 (dd, $J_1$ = 8.2, $J_2$ = 3.5 Hz, 4H), 2.42 (s, 6H). $^{19}F$ NMR (565 MHz, $CDCl_3$, 25 °C): δ –74.4 (d, $J$ = 1014 Hz). $^{31}P$ NMR (243 MHz, $CDCl_3$, 25 °C): δ 42.1 (d, $J$ = 1015 Hz). Spectroscopic data matched those previously reported in literature.[36]

bis(4-fluorophenyl)phosphinic fluoride (**7c**)

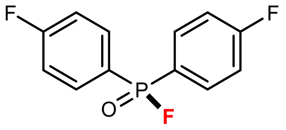

Prepared according to general procedure C for phosphinic acids and hydrogenphosphates. Yield was determined with $^1$H NMR with naphthalene as an internal standard (25.62 mg, >99%). $^1$H NMR (600 MHz, $CDCl_3$, 25 °C): δ 7.85 (m, 4H), 7.23 (m, 4H). $^{19}$F NMR (565 MHz, $CDCl_3$, 25 °C): δ –72.98 (d, *J* = 1019 Hz, 1F), –103.3 (m, 2F). $^{31}$P NMR (243 MHz, $CDCl_3$, 25 °C): δ 38.9 (d, *J* = 1005 Hz). Spectroscopic data matched those previously reported in literature.[37]

bis(4-nitrophenyl)phosphinic fluoride (**7d**)

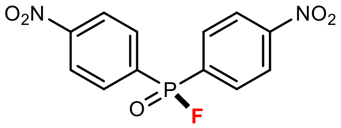

Prepared according to general procedure C for phosphinic acids and hydrogenphosphates. Yield was determined with $^1$H NMR with naphthalene as an internal standard (27.30 mg, 88%). $^1$H NMR (600 MHz, $CDCl_3$, 25 °C): δ 8.41 (dd, $J_1$ = 8.7, $J_2$ = 3.0 Hz, 4H), 8.08 (dd, $J_1$ = 12.6, $J_2$ = 8.7 Hz, 4H). $^{19}$F NMR (565 MHz, $CDCl_3$, 25 °C): δ –74.3 (d, *J* = 1039 Hz). $^{31}$P NMR (243 MHz, $CDCl_3$, 25 °C): δ 34.5 (d, *J* = 1043 Hz). HRMS (ESI-TOF): m/z $[M+H]^+$ calculated for $C_{12}H_8FN_2O_5P$: 311.0233; found: 311.0226

Due to potential severe toxicity of previously uncharacterized fluorophosphates we abstained from isolation of this product and its full characterization. NMR data is given for reaction mixtures.

bis(4-methoxyphenyl)phosphinic fluoride (**7e**)

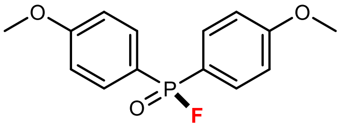

Prepared according to general procedure C for phosphinic acids and hydrogenphosphates. Yield was determined with $^1$H NMR with naphthalene as an internal standard (25.22 mg, 90%). $^1$H NMR (600 MHz, $CDCl_3$, 25 °C): δ 7.74 (m, 4H), 7.00 (dd, $J_1$ = 8.8, $J_2$ = 3.0 Hz, 4H), 3.86 (s, 6H). $^{19}$F NMR (565 MHz, $CDCl_3$, 25 °C): δ –72.6 (d, *J* = 1007 Hz). $^{31}$P NMR (243 MHz, $CDCl_3$, 25 °C): δ 42.1 (d, *J* = 1010 Hz). Spectroscopic data matched those previously reported in literature.[36]

diethyl phosphorofluoridate (**7f**)

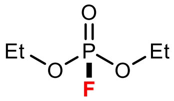

Prepared according to general procedure C for phosphinic acids and hydrogenphosphates. Yield was determined with $^{1}$H NMR with naphthalene as an internal standard (13.58 mg, 87%). $^{1}$H NMR (600 MHz, $CDCl_3$, 25 °C): δ 4.26 (q, $J$ = 7.1 Hz, 4H), 1.40 (t, $J$ = 7.1 Hz, 6H). $^{19}$F NMR (565 MHz, $CDCl_3$, 25 °C): δ –80.9 (d, $J$ = 978 Hz). $^{31}$P NMR (243 MHz, $CDCl_3$, 25 °C): δ –9.1 (d, $J$ = 969 Hz). Spectroscopic data matched those previously reported in literature.[36]

dibutyl phosphorofluoridate (**7g**)

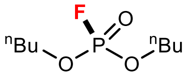

Prepared according to general procedure C for phosphinic acids and hydrogenphosphates. Yield was determined with $^{1}$H NMR with naphthalene as an internal standard (20.37 mg, 96%). $^{1}$H NMR (600 MHz, $CDCl_3$, 25 °C): δ 4.19 (q, $J$ = 6.7 Hz, 4H), 1.74–1.68 (m, 4H), 1.49–1.39 (m, 4H), 0.95 (t, $J$ = 7.4 Hz, 6H). $^{19}$F NMR (565 MHz, $CDCl_3$, 25 °C): δ –81.6 (d, $J$ = 978 Hz). $^{31}$P NMR (243 MHz, $CDCl_3$, 25 °C): δ –8.9 (dd, $J_1$ = 978, $J_2$ = 8.2 Hz). Spectroscopic data matched those previously reported in literature.[38]

bis(2-ethylhexyl) phosphorofluoridate (**7h**)

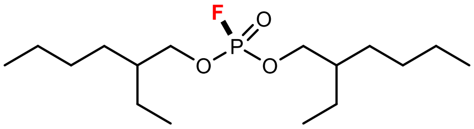

Prepared according to general procedure C for phosphinic acids and hydrogenphosphates. Yield was determined with $^{1}$H NMR with naphthalene as an internal standard (32.44 mg, >99%). $^{1}$H NMR (600 MHz, $CDCl_3$, 25 °C): δ 4.14–4.06 (m, 4H), 1.65–1.58 (m, 2H), 1.44–1.28 (m, 16H), 0.95–0.87 (m, 12H). $^{19}$F NMR (565 MHz, $CDCl_3$, 25 °C): δ –82.3 (dt, $J_1$ = 977, $J_2$ = 10.9 Hz). $^{31}$P NMR (243 MHz, $CDCl_3$, 25 °C): δ –8.6 (d, $J$ = 976 Hz). Spectroscopic data matched those previously reported in literature.[38]

bis(4-nitrophenyl)phosphinic fluoride (**7d**)

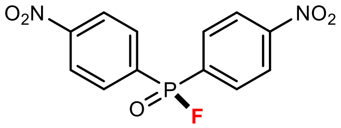

**Due to potential severe toxicity of previously uncharacterized fluorophosphates we abstained from isolation of this product and its full characterization. NMR data is given for reaction mixtures.**

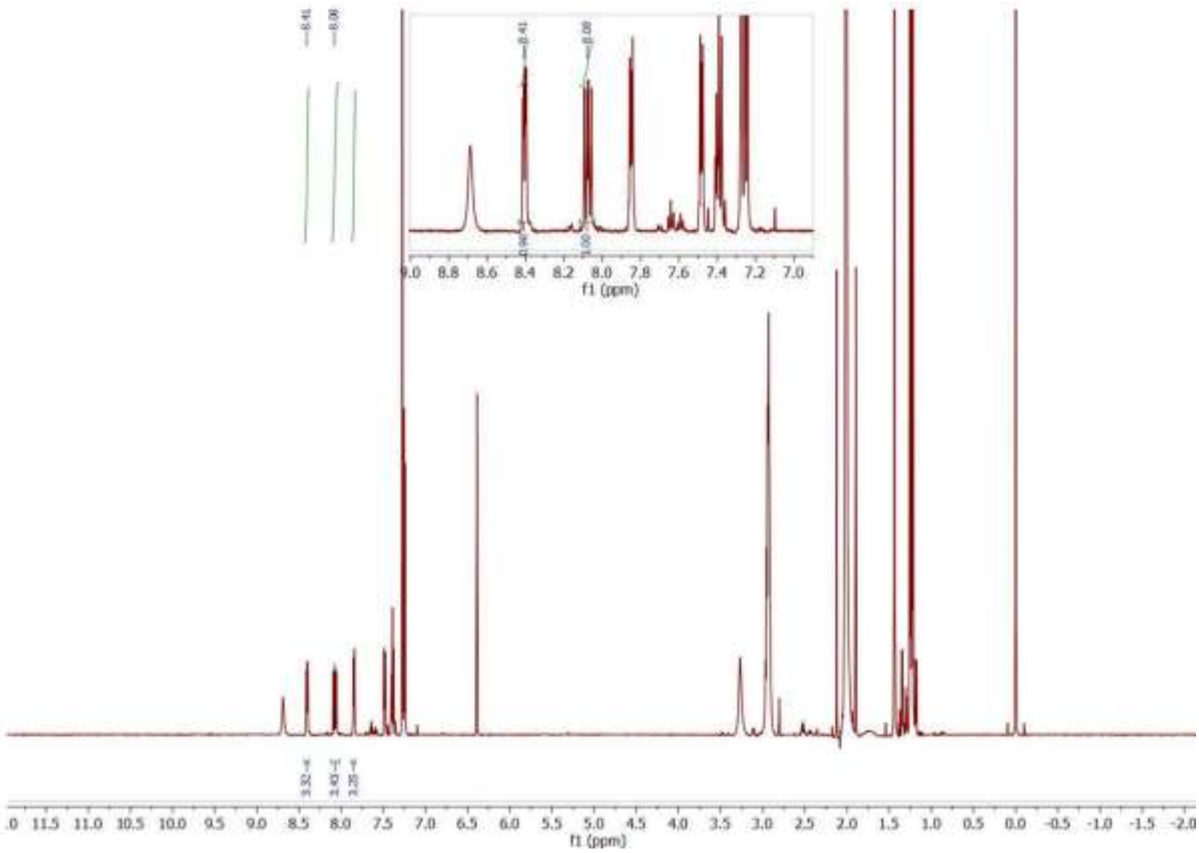

**Figure S- 4:** $^1$H NMR (600 MHz, $CDCl_3$) spectrum of reaction mixture with **7d.**

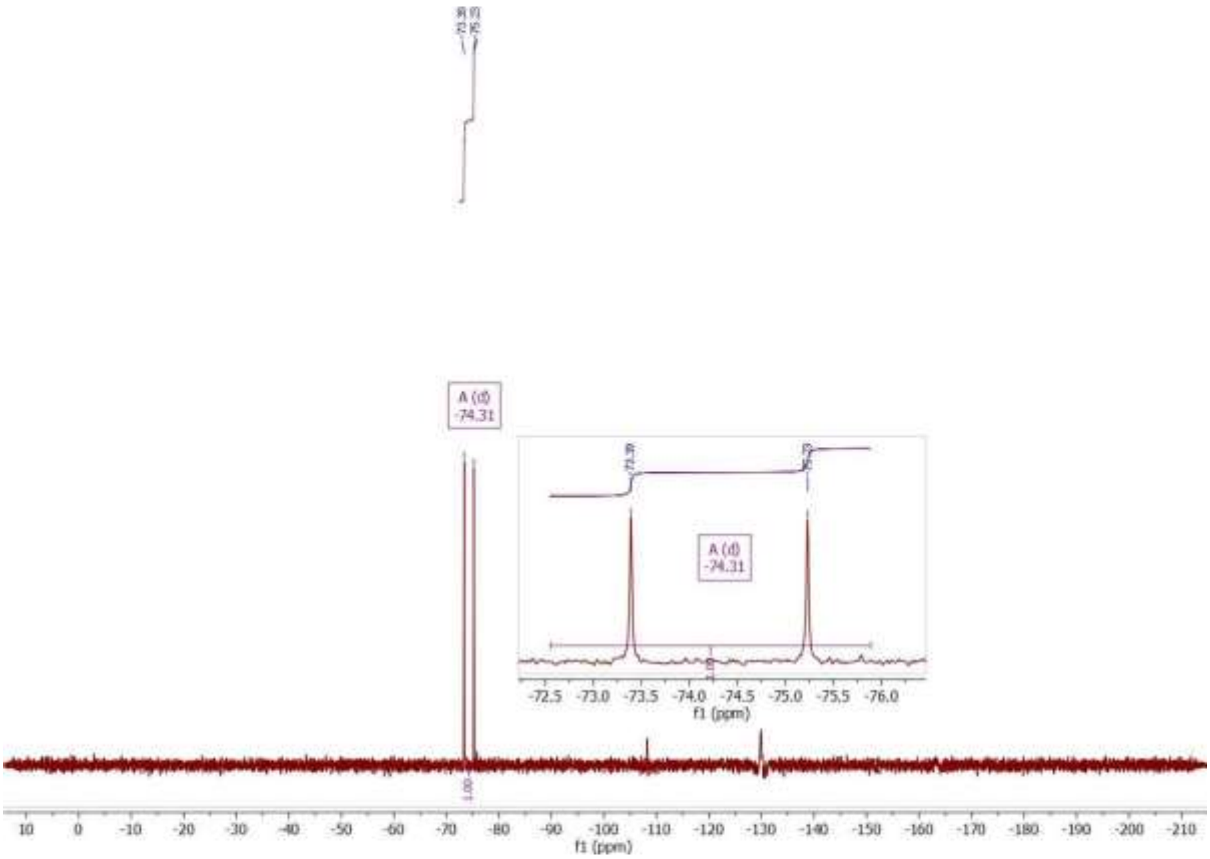


**Figure S- 5:** $^{19}F$ NMR (565 MHz, $CDCl_3$) spectrum of reaction mixture with **7d**.

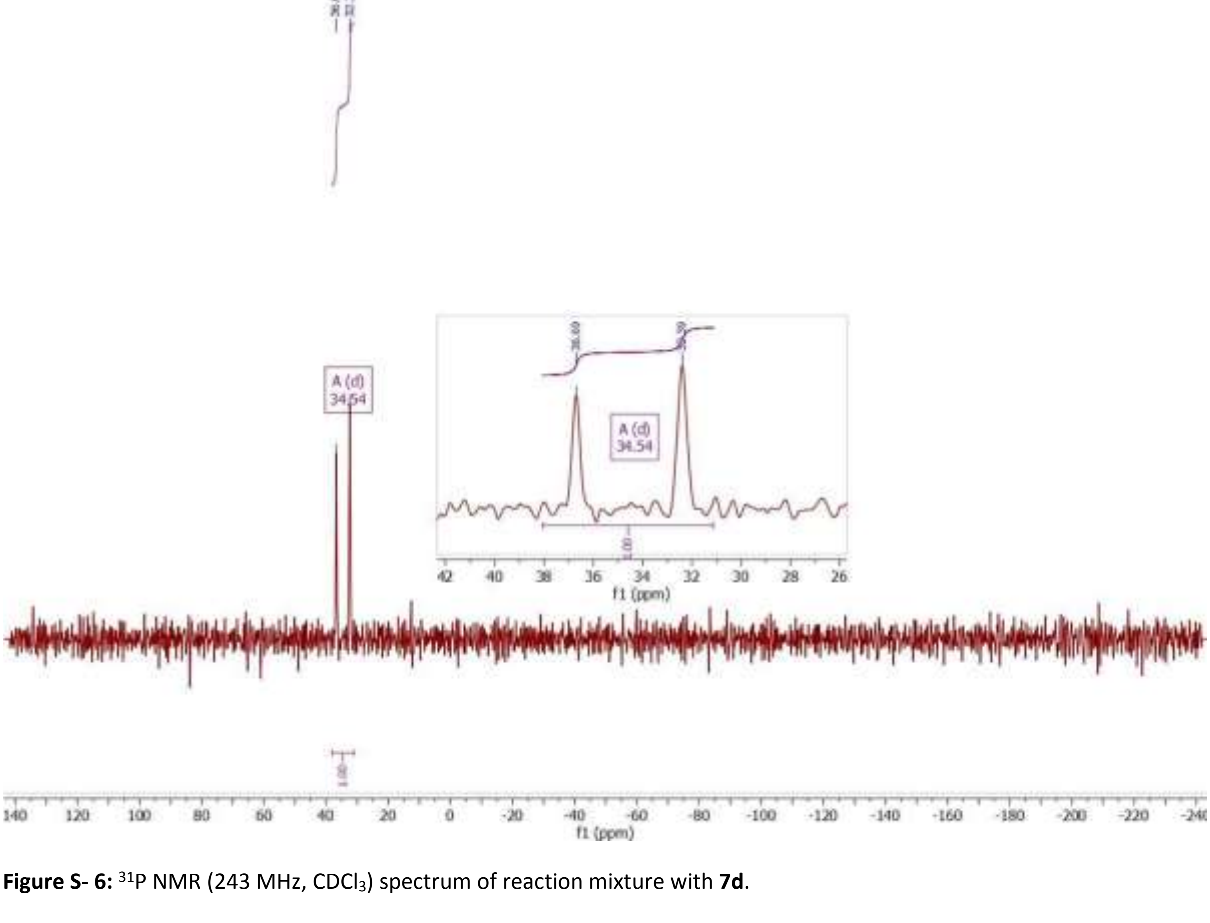


**Figure S- 6:** $^{31}P$ NMR (243 MHz, $CDCl_3$) spectrum of reaction mixture with **7d**.

4–trifluoromethylthiobenzyl fluoride (**3k**)

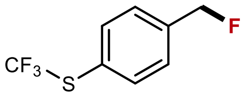

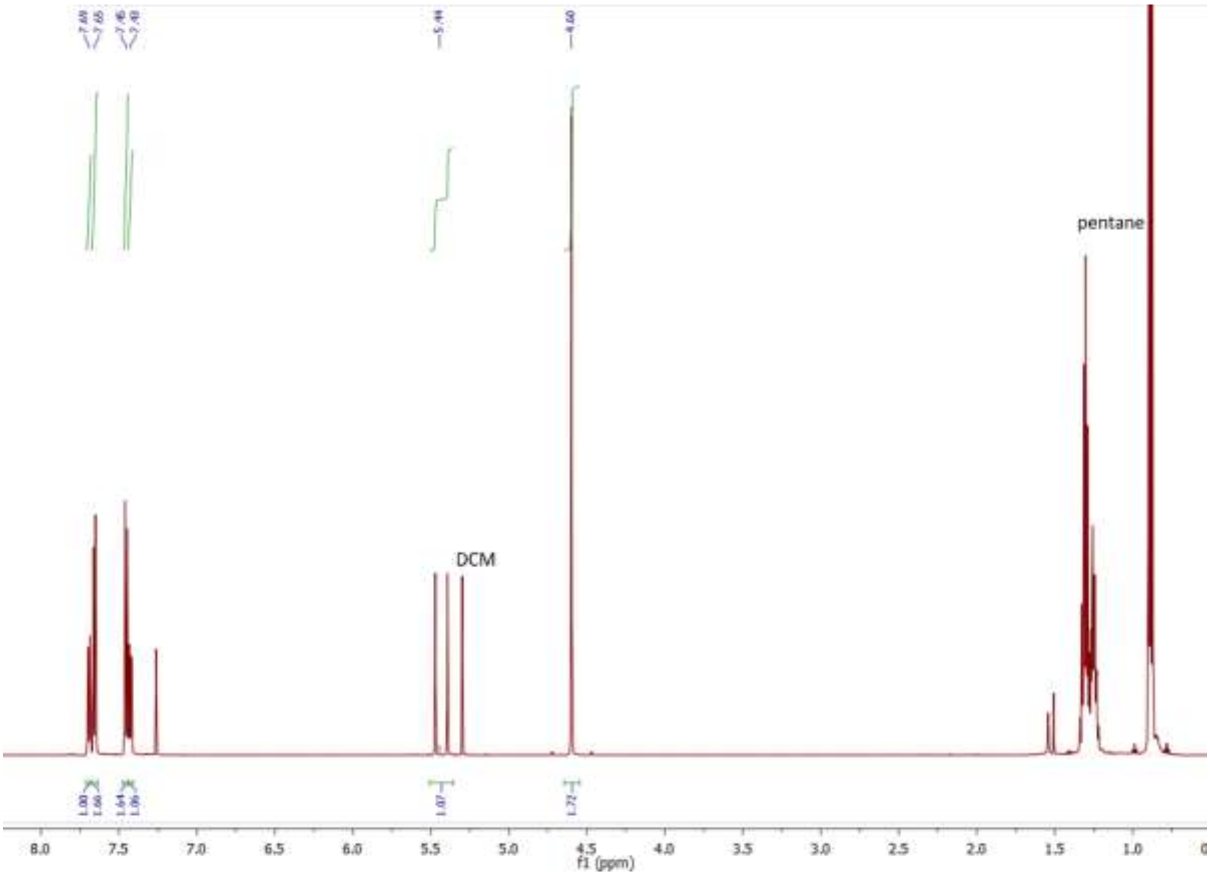


**Figure S- 4:** $^{1}$H NMR (600 MHz, $CDCl_3$) spectrum of mixture of **3k** and its corresponding chloride 1:1.7.

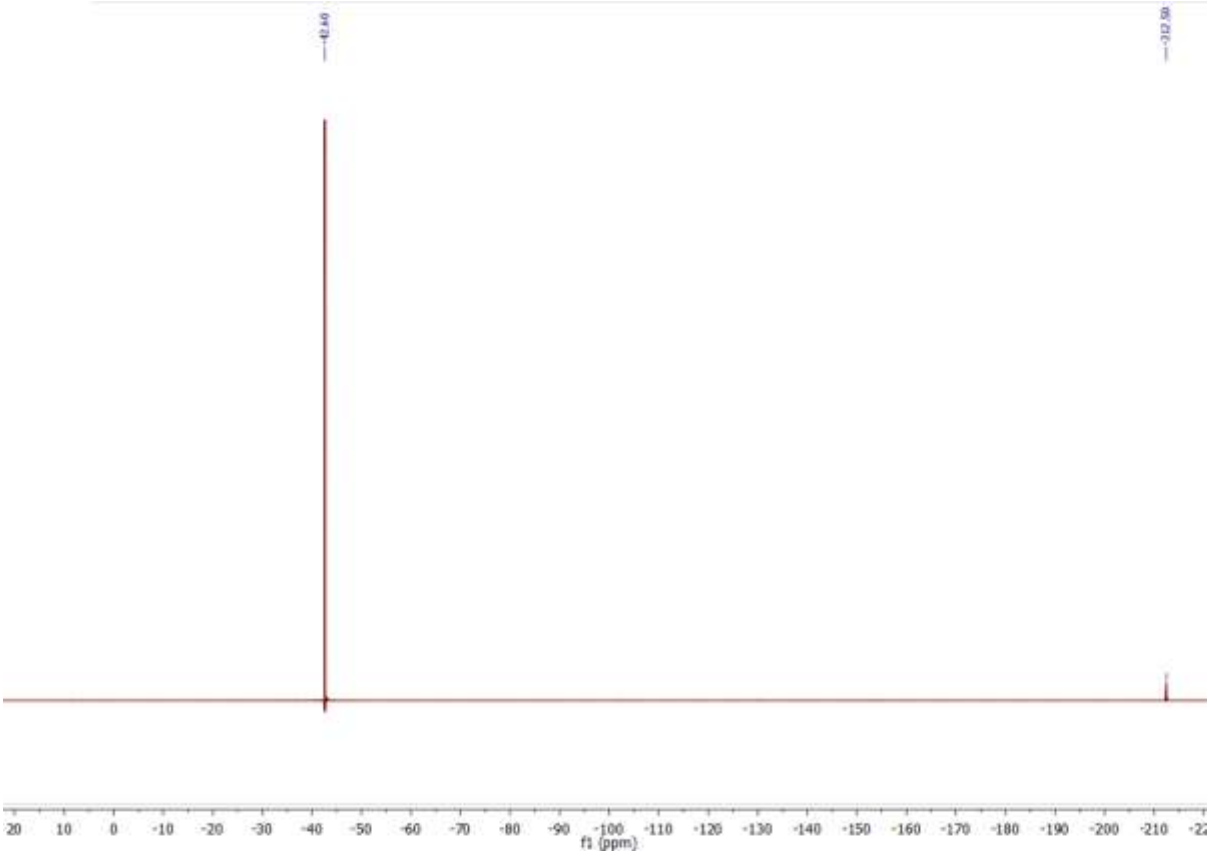


**Figure S- 5:** $^{19}F$ (565 MHz, $CDCl_3$) NMR spectrum of mixture of **3k** and its corresponding chloride 1:1.7.

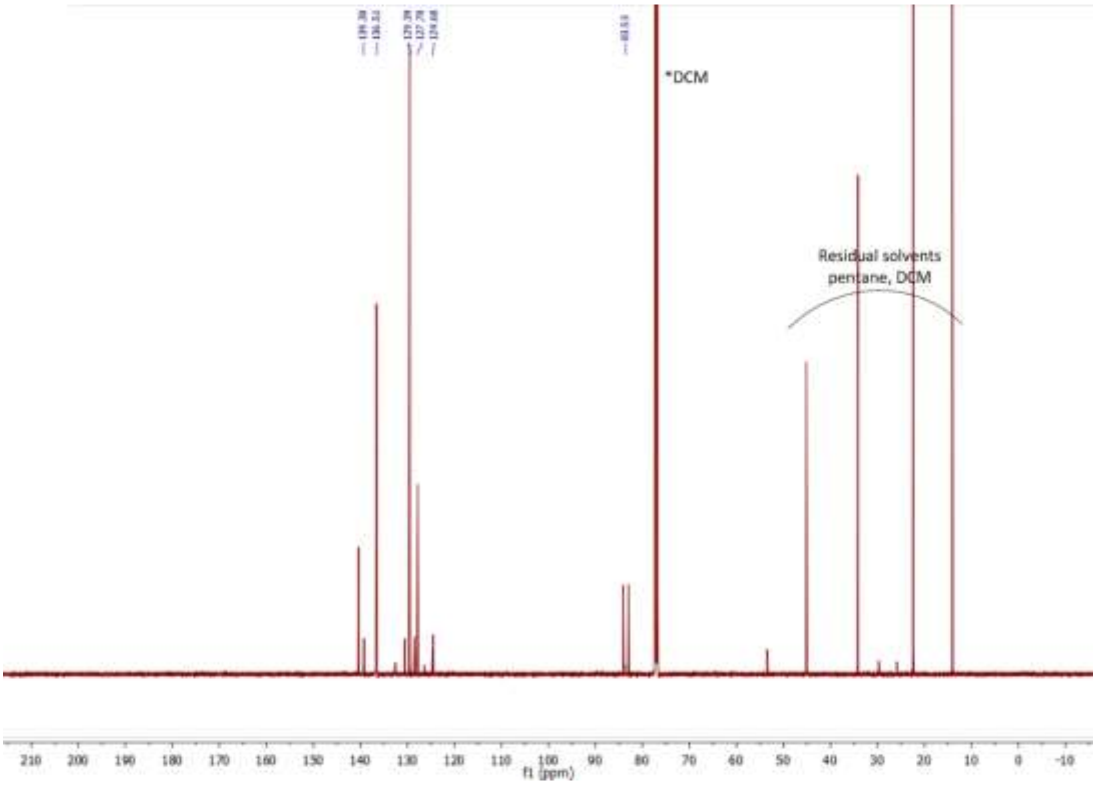


**Figure S- 6:** $^{13}C\{^1H\}$ NMR (151 MHz, $CDCl_3$) spectrum of mixture of **3k** and its corresponding chloride 1:1.7[1].

[1] Discrepancy in the ratio is due to volatility of the products during isolation.

## References


1. Jelen, J.; Tavčar, G., Deoxyfluorination of Electron-Deficient Phenols. *Org. Lett.* **2023,** *25*, 3649-3653.
2. Botez, L.; de Jong, G. B.; Slootweg, J. C.; Deelman, B.-J., A Direct Catalytic Synthesis of Sodium Diarylphosphinates and Their Corresponding Acids from Sodium Phosphinate. *Eur. J. Org. Chem.* **2017,** *2017*, 434-437.
3. Bloom, S.; McCann, M.; Lectka, T., Photocatalyzed Benzylic Fluorination: Shedding “Light” on the Involvement of Electron Transfer. *Org. Lett.* **2014,** *16*, 6338–6341.
4. An, L.; Xiao, Y.-L.; Min, Q.-Q.; Zhang, X., Facile Access to Fluoromethylated Arenes by Nickel-Catalyzed Cross-Coupling between Arylboronic Acids and Fluoromethyl Bromide. *Angew. Chem. Int. Ed.* **2015,** *54*, 9079-9083.
5. Yadav, A. K.; Srivastava, V. P.; Yadav, L. D. S., An easy access to fluoroalkanes by deoxygenative hydrofluorination of carbonyl compounds via their tosylhydrazones. *Chem. Comm.* **2013,** *49*, 2154-2156.
6. Goldberg, N. W.; Shen, X.; Li, J.; Ritter, T., AlkylFluor: Deoxyfluorination of Alcohols. *Org. Lett.* **2016,** *18*, 6102-6104.
7. Bromilow, J.; Brownlee, R. T. C.; Page, A. V., The origin of fluorine-19 and carbon-13 substituent chemical shifts in substituted benzyl fluorides. *Tetrahedron Lett.* **1976,** *17*, 3055-3058.
8. Rae, I. D.; Burgess, D. A.; Bombaci, S.; Baron, M. L.; Woolcock, M. L., Fluorine magnetic resonance studies. VI. Fluorine-fluorine coupling over four bonds in α-Fluoro- and α,α-Difluoro-toluenes. *Aust. J. Chem.* **1984,** *37*, 1437-1446.
9. Champagne, P. A.; Benhassine, Y.; Desroches, J.; Paquin, J.-F., Friedel–Crafts Reaction of Benzyl Fluorides: Selective Activation of C⍰F Bonds as Enabled by Hydrogen Bonding. *Angew. Chem. Int. Ed.* **2014,** *53*, 13835-13839.
10. Chen, J.; Lin, J.-H.; Xiao, J.-C., Halogenation through Deoxygenation of Alcohols and Aldehydes. *Org. Lett.* **2018,** *20*, 3061-3064.
11. Alič, B.; Petrovčič, J.; Jelen, J.; Tavčar, G.; Iskra, J., Renewable Reagent for Nucleophilic Fluorination. *J. Org. Chem.* **2022,** *87*, 5987-5993.
12. Dempsey, K.; Mir, R.; Smajlagic, I.; Dudding, T., Expanding the repertoire of cyclopropenium ion phase transfer catalysis: Benzylic fluorination. *Tetrahedron* **2018,** *74*, 3507-3511.
13. Ma, J.-j.; Yi, W.-b.; Lu, G.-p.; Cai, C., Transition-metal-free C–H oxidative activation: persulfate-promoted selective benzylic mono- and difluorination. *Org. Biomol. Chem.* **2015,** *13*, 2890-2894.
14. Madani, A.; Anghileri, L.; Heydenreich, M.; Möller, H. M.; Pieber, B., Benzylic Fluorination Induced by a Charge-Transfer Complex with a Solvent-Dependent Selectivity Switch. *Org. Lett.* **2022,** *24*, 5376-5380.
15. Kim, D. W.; Jeong, H.-J.; Lim, S. T.; Sohn, M.-H., Tetrabutylammonium Tetra(tert-Butyl Alcohol)-Coordinated Fluoride as a Facile Fluoride Source. *Angew. Chem. Int. Ed.* **2008,** *47*, 8404-8406.
16. Mirabdolbaghi, R.; Dudding, T.; Stamatatos, T., A Class of Phase-Transfer Catalyst with Interionic Strain: Insight into the Bonding of Disubstituted N- vs Carbene-Stabilized NI-Centered Cations. *Org. Lett.* **2014,** *16*, 2790-2793.
17. Nielsen, M. K.; Ahneman, D. T.; Riera, O.; Doyle, A. G., Deoxyfluorination with Sulfonyl Fluorides: Navigating Reaction Space with Machine Learning. *J. Am. Chem. Soc.* **2018,** *140*, 5004-5008.
18. Zeng, J.; Vedachalam, S.; Xiang, S.; Liu, X.-W., Direct C-Glycosylation of Organotrifluoroborates with Glycosyl Fluorides and Its Application to the Total Synthesis of (+)-Varitriol. *Org. Lett.* **2011,** *13*, 42-45.

19. Munoz, S. B.; Dang, H.; Ispizua-Rodriguez, X.; Mathew, T.; Prakash, G. K. S., Direct Access to Acyl Fluorides from Carboxylic Acids Using a Phosphine/Fluoride Deoxyfluorination Reagent System. *Org. Lett.* **2019,** *21*, 1659-1663.
20. Liang, Y.; Zhao, Z.; Taya, A.; Shibata, N., Acyl Fluorides from Carboxylic Acids, Aldehydes, or Alcohols under Oxidative Fluorination. *Org. Lett.* **2021,** *23*, 847-852.
21. Arisawa, M.; Yamada, T.; Yamaguchi, M., Rhodium-catalyzed interconversion between acid fluorides and thioesters controlled using heteroatom acceptors. *Tetrahedron Lett.* **2010,** *51*, 6090-6092.
22. Birrell, J. A.; Desrosiers, J.-N.; Jacobsen, E. N., Enantioselective Acylation of Silyl Ketene Acetals through Fluoride Anion-Binding Catalysis. *J. Am. Chem. Soc.* **2011,** *133*, 13872-13875.
23. Chambers, R. D.; Sandford, G.; Trmcic, J.; Okazoe, T., Elemental Fluorine. Part 21.1 Direct Fluorination of Benzaldehyde Derivatives. *Org. Process Res. Dev.* **2008,** *12*, 339-344.
24. Tryniszewski, M.; Barbasiewicz, M., Gram-Scale Preparation of Acyl Fluorides and Their Reactions with Hindered Nucleophiles. *Synthesis* **2021,** *54*, 1446-1460.
25. Gonay, M.; Batisse, C.; Paquin, J.-F., Synthesis of Acyl Fluorides from Carboxylic Acids Using NaF-Assisted Deoxofluorination with XtalFluor-E. *J. Org. Chem.* **2020,** *85*, 10253-10260.
26. Boreux, A.; Indukuri, K.; Gagosz, F.; Riant, O., Acyl Fluorides as Efficient Electrophiles for the Copper-Catalyzed Boroacylation of Allenes. *ACS Catal.* **2017,** *7*, 8200-8204.
27. Vogel, J. A.; Hammami, R.; Ko, A.; Datta, H.; Eiben, Y. N.; Labenne, K. J.; McCarver, E. C.; Yilmaz, E. Z.; Melvin, P. R., Synthesis of Highly Reactive Sulfone Iminium Fluorides and Their Use in Deoxyfluorination and Sulfur Fluoride Exchange Chemistry. *Org. Lett.* **2022,** *24*, 5962-5966.
28. Matsumoto, A.; Wang, Z.; Maruoka, K., Radical-Mediated Activation of Esters with a Copper/Selectfluor System: Synthesis of Bulky Amides and Peptides. *J. Org. Chem.* **2021,** *86*, 5401-5411.
29. Arisawa, M.; Igarashi, Y.; Kobayashi, H.; Yamada, T.; Bando, K.; Ichikawa, T.; Yamaguchi, M., Equilibrium shift in the rhodium-catalyzed acyl transfer reactions. *Tetrahedron* **2011,** *67*, 7846-7859.
30. Scattolin, T.; Deckers, K.; Schoenebeck, F., Direct Synthesis of Acyl Fluorides from Carboxylic Acids with the Bench-Stable Solid Reagent (Me4N)SCF3. *Org. Lett.* **2017,** *19*, 5740-5743.
31. Suaifan, G. A. R. Y.; Mahon, M. F.; Arafat, T.; Threadgill, M. D., Effects of steric bulk and stereochemistry on the rates of diketopiperazine formation from N-aminoacyl-2,2-dimethylthiazolidine-4-carboxamides (Dmt dipeptide amides)—a model for a new prodrug linker system. *Tetrahedron* **2006,** *62*, 11245-11266.
32. Colombo, R.; Wang, Z.; Han, J.; Balachandran, R.; Daghestani, H. N.; Camarco, D. P.; Vogt, A.; Day, B. W.; Mendel, D.; Wipf, P., Total Synthesis and Biological Evaluation of Tubulysin Analogues. *J. Org. Chem.* **2016,** *81*, 10302-10320.
33. Carpino, L. A.; Mansour, E.-S. M. E.; Sadat-Aalaee, D., tert-Butyloxycarbonyl and benzyloxycarbonyl amino acid fluorides. New, stable rapid-acting acylating agents for peptide synthesis. *J. Org. Chem.* **1991,** *56*, 2611-2614.
34. Brittain, W. D. G.; Cobb, S. L., Carboxylic Acid Deoxyfluorination and One-Pot Amide Bond Formation Using Pentafluoropyridine (PFP). *Org. Lett.* **2021,** *23*, 5793-5798.
35. Malapit, C. A.; Bour, J. R.; Laursen, S. R.; Sanford, M. S., Mechanism and Scope of Nickel-Catalyzed Decarbonylative Borylation of Carboxylic Acid Fluorides. *J. Am. Chem. Soc.* **2019,** *141*, 17322-17330.
36. Eljo, J.; Murphy, G., Direct, Oxidative Halogenation of Diaryl- or Dialkylphosphine oxides with (Dihaloiodo)arenes. *Tetrahedron Lett.* **2018,** *59*, 2965-2969.
37. Chen, Q.; Zeng, J.; Yan, X.; Huang, Y.; Wen, C.; Liu, X.; Zhang, K., Electrophilic Fluorination of Secondary Phosphine Oxides and Its Application to P–O Bond Construction. *J. Org. Chem.* **2016,** *81*, 10043-10048.
38. Li, Q.-W.; Zhang, X.-Y.; Lu, L.; Wu, Z.-Q.; Li, J.; Li, G.-Z.; Sun, K.; Yang, S.-D.; Yang, B., TFAA/DMSO-Promoted Fluorination of P(O)−H and P(O)−OH Compounds: Compatible Access to Fluorophosphonates and Phosphonofluoridates. *Adv. Synth. Catal.* **2022,** *364*, 938-946.
